# OVERTURE: A Worldwide, Prospective, Observational Study of Disease Characteristics in Patients With ADPKD

**DOI:** 10.1016/j.ekir.2023.02.1073

**Published:** 2023-02-13

**Authors:** Ronald D. Perrone, Dorothee Oberdhan, John Ouyang, Daniel G. Bichet, Klemens Budde, Arlene B. Chapman, Berenice Y. Gitomer, Shigeo Horie, Albert C.M. Ong, Vicente E. Torres, A. Neil Turner, Holly Krasa

**Affiliations:** 1 Division of Nephrology, Tufts Medical Center, Tufts University School of Medicine, Boston, Massachusetts, USA; 2 Otsuka Pharmaceutical Development & Commercialization, Inc., Rockville, Maryland, USA; 3 Division of Nephrology, Département de Médecine, Pharmacologie et Physiologie, Hôpital du Sacré-Cœur de Montréal, Université de Montréal, Quebec, Canada; 4 Charité-Universitätsmedizin Berlin, Department of Nephrology and Medical Intensive Care, Berlin, Germany; 5 Section of Nephrology, University of Chicago School of Medicine, Chicago, Illinois, USA; 6 Department of Medicine, Division of Renal Diseases and Hypertension, University of Colorado Anschutz Medical Campus, Aurora, Colorado, USA; 7 Department of Urology, Juntendo University Graduate School of Medicine, Tokyo, Japan; 8 Kidney Genetics Group, Academic Nephrology Unit, Department of Infection, Immunity and Cardiovascular Disease, University of Sheffield Medical School, Sheffield, UK; 9 Division of Nephrology and Hypertension, Mayo Clinic, Rochester, Minnesota, USA; 10 Renal and Autoimmunity Group, MRC Center for Inflammation, University of Edinburgh, Edinburgh, UK; 11 Blue Persimmon Group LLC, Washington, District of Columbia, USA

**Keywords:** autosomal dominant polycystic kidney disease, cohort, disease progression, natural history, observational, patient-reported outcomes

## Abstract

**Introduction:**

The course of autosomal dominant polycystic kidney disease (ADPKD) varies greatly among affected individuals, necessitating natural history studies to characterize the determinants and effects of disease progression. Therefore, we conducted an observational, longitudinal study (OVERTURE; NCT01430494) of patients with ADPKD.

**Methods:**

This prospective study enrolled a large international population (*N* = 3409) encompassing a broad spectrum of ages (12–78 years), chronic kidney disease (CKD) stages (G1–G5), and Mayo imaging classifications (1A–1E). Outcomes included kidney function, complications, quality of life, health care resource utilization, and work productivity.

**Results:**

Most subjects (84.4%) completed ≥12 months of follow-up. Consistent with earlier findings, each additional l/m of height-adjusted total kidney volume (htTKV) on magnetic resonance imaging (MRI) was associated with worse outcomes, including lower estimated glomerular filtration rate (eGFR) (regression coefficient 17.02, 95% confidence interval [CI] 15.94–18.11) and greater likelihood of hypertension (odds ratio [OR] 1.25, 95% CI 1.17–1.34), kidney pain (OR 1.22, 95% CI 1.11–1.33), and hematuria (OR 1.35, 95% CI 1.21–1.51). Greater baseline htTKV was also associated with worse patient-reported health-related quality of life (e.g., ADPKD Impact Scale physical score, regression coefficient 1.02, 95% CI 0.65–1.39), decreased work productivity (e.g., work days missed, regression coefficient 0.55, 95% CI 0.18–0.92), and increased health care resource utilization (e.g., hospitalizations, OR 1.48, 95% CI 1.33–1.64) during follow-up.

**Conclusion:**

Although limited by a maximum 3-year duration of follow-up, this observational study characterized the burden of ADPKD in a broad population and indicated the predictive value of kidney volume for outcomes other than kidney function.

## Graphical abstract

**Figure:**
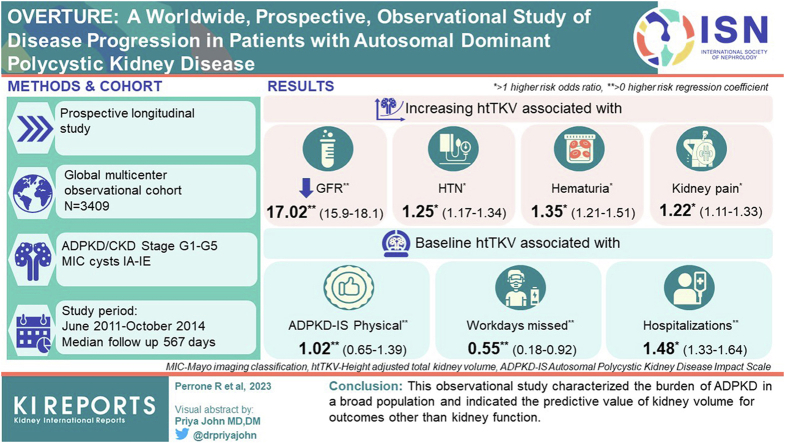

See Commentary on Page 951

The complications and symptoms of ADPKD develop gradually, typically over the course of decades. Manifestations of ADPKD are phenotypically heterogeneous, with high interindividual variability, both in speed of disease progression and in clinical presentation.^1^ Questions regarding medical management at an individual patient level have therefore been controversial, including how to counsel patients with ADPKD, the appropriate frequency of monitoring, and whom to select for treatment.^2^ Moreover, the genetic determinants of ADPKD progression are complex, making predictive genetic testing challenging and expensive.^2^^,^^3^

Longitudinal, observational studies are necessary to better characterize the clinical course of ADPKD and identify patient subgroups that may require differentiated medical management, and to estimate the health economic consequences of ADPKD to the individual and to society. The Consortium for Radiologic Imaging Studies of Polycystic Kidney Disease (CRISP), an ongoing, prospective, longitudinal, observational study that enrolled 241 subjects, has demonstrated the value of such research, notably by establishing baseline htTKV as a predictor of CKD progression in ADPKD.^4^ Data from CRISP have been used to develop risk stratification models for patients with ADPKD.^5^^,^^6^

Whereas the CRISP cohort,^7^ along with smaller prospective cohort studies,^8^^,^^9^ and patient registries^10^, ^11^, ^12^, ^13^ have addressed questions regarding ADPKD progression and outcomes, there remains a need for longitudinal data reflecting the wide spectrum of age-related CKD stages encountered in the ADPKD population. Inclusion criteria for CRISP were designed to specifically enroll a population of young subjects (ages: 15–45 years) with relatively preserved kidney function (predominantly CKD stages G1–G2) and enriched with known risk factors for progressive kidney disease.^4^^,^^14^ However, patients with ADPKD at later stages of CKD may progress differently than earlier-stage patients.^15^ Interventional clinical trials of tolvaptan, TEMPO 3:4 (NCT00428948) and REPRISE (NCT02160145), enrolled subjects with ADPKD at CKD stages G1–G3 and G2–G4, respectively, with elevated risk of rapid progression.^16^^,^^17^ The interpretation and application of the trial findings to clinical practice would benefit from context on how the enrolled subjects reflected the overall ADPKD population.

Observational studies should account for the impact of ADPKD progression on outcomes broadly understood, that is, ADPKD-related clinical outcomes other than kidney function decline, and health economic considerations. Although ADPKD leads to lifetime treatment costs and disease burden, there have been few studies of ADKPD-specific health care resource utilization and medical costs.^18^^,^^19^ Likewise, the effects of ADPKD on work status and productivity have not been established.

Accordingly, we undertook OVERTURE, a prospective, large-scale, global, observational study, to identify factors that predict progression to ADPKD-related outcomes (e.g., decline in kidney function, kidney pain, hematuria, infection) in a broadly representative global population. Another objective was to explore the magnitude and determinants of the health economic effects of ADPKD, including the impact of disease on health care resource utilization and productivity.

## Methods

### Study Design

OVERTURE (ClinicalTrials.gov identifier: NCT01430494) was a global, multicenter, observational, longitudinal study to assess the rates, characteristics, and determinants of disease progression in subjects with ADPKD treated in routine clinical care. Because this study was observational, there were no investigational therapies; subjects were treated by their individually chosen physicians according to their physicians’ chosen standard of care.

The study was conducted at 285 sites in the following 20 countries: Argentina, Australia, Belgium, Brazil, Canada, Czech Republic, France, Germany, Italy, Japan, Netherlands, Norway, Poland, Romania, Spain, Sweden, Switzerland, Turkey, United Kingdom, and the United States. A list of study sites and principal investigators is provided in the Supplementary Material. The study was initiated in June 2011 and completed in October 2014.

### Participants

The study enrolled female and male subjects aged 12 to 70 years, inclusive, who had been diagnosed with ADPKD by modified Ravine criteria (Supplementary Table S1).^20^ To participate in OVERTURE, subjects were required to have htTKV ≥300 ml/m height by ultrasound (within 1 year prior to baseline) or ≥250 ml/m height by MRI (within 1 year prior to baseline). Individuals who had any medical condition that could interfere with evaluation of the study objectives (e.g., inability to comply with MRI) were excluded. Similarly, patients undergoing any current or expected (within the next 6 months) interventions for the treatment of ADPKD affecting kidney volume were excluded without the prior approval of the sponsor. Initially, study sites inadvertently excluded subjects on dialysis or those who had kidney transplants due to a misinterpretation of the protocol. Subjects in these categories were eventually enrolled in the study, resulting in a small population who were on dialysis or post-transplantation.

### Study Assessments

Study visits took place at baseline, month 6, month 12, month 18, and every 6 months thereafter for up to 36 months. Subjects were expected to be followed for a minimum of 12 months and until the objectives of the program were achieved. At the baseline visit, subjects provided a detailed general medical and ADPKD history. Abdominal girth; blood pressure; height; weight; and samples for blood and urine creatinine, urine albumin, and urine osmolality were collected at each visit. Additional laboratory results potentially relevant to the progression of ADPKD, obtained as part of standard of care, were collected. Collection of cystatin-C was not included in the protocol. Outcomes related to ADPKD (e.g., significant drop in kidney function, liver or other cysts, hypertension, kidney pain, hematuria, nephrolithiasis, and infection), health care resource utilization (hospitalizations, nonhospitalization visits, procedures), use of concomitant medications, patient-reported outcome measures, and impact on work and productivity were collected. Significant drop in kidney function was defined as dialysis, transplant, doubling of serum creatinine from baseline, or other significant drop as determined by the investigator.

An MRI to determine TKV was performed at baseline and between months 11 to 19 after enrollment, with the MRI done as close to a scheduled clinic visit as possible. All MRI assessments were performed according to a study-specific imaging protocol, with images read centrally to provide consistent data and evaluation methodology across all subjects. To estimate kidney function, the 2009 CKD Epidemiology Collaboration Formula was used for all subjects (with adjustment for race and Japanese ethnicity).^21^

Patient-reported outcome assessment instruments used were the ADPKD Impact Scale (ADPKD-IS), ADPKD Urinary Impact Scale, the Brief Pain Inventory-Short Form, the Short Form 12-item Health Survey version 2 (SF-12v2), and the EQ-5D-3L. The ADPKD-IS measures ADPKD-related symptom burden over the past 2 weeks in 3 domains (Physical, Emotional, and Fatigue).^22^ The ADPKD Urinary Impact Scale measures ADPKD-related urinary symptom burden over the last week covering 3 domains (daytime urinary urgency, daytime urinary frequency, and nocturia).^23^ The Brief Pain Inventory-Short Form is a self-administered generic questionnaire used to evaluate the severity of a subject’s pain and the impact of this pain on the subject’s daily functioning.^24^ The SF-12v2 assesses generic health-related quality of life in the past month within 2 broad domains, the Physical Component Summary and Mental Component Summary scores.^25^ The EQ-5D-3L is a generic instrument that assesses 5 health dimensions: mobility, self-care, usual activities (work, study, housework, family, or leisure), pain or discomfort, and anxiety or depression.^26^

### Study Objectives

The primary objective of this study was to investigate the association of TKV with ADPKD-related outcomes. Secondary objectives focused on the impact of ADPKD on disease-specific patient-reported outcomes, general quality of life, health care resource utilization, and productivity. Exploratory objectives included identifying additional clinical determinants and predictors of disease progression.

### Statistical Analysis

#### Sample Size

No formal sample size estimation was performed; the 3000-subject target sample size was based on the number expected to yield appropriate population-based, cross-sectional information. A minimum of approximately 2500 subjects who were between the ages of 16 to 48 years, had eGFR ≥45 ml/min per 1.73 m^2^, and who had htTKV by MRI ≥400 ml/m were targeted. The purpose of this enrollment strategy was to enrich the study population with subjects likely to benefit from disease-modifying therapy, thereby potentially informing the design of future interventional clinical trials.

#### Analyses of Study Outcomes

Summary statistics were calculated for subject disposition and baseline characteristics. Associations between baseline htTKV and ADPKD-related and health care resource utilization outcomes during the study were explored using linear and generalized mixed models accounting for repeated measures by study visit. All models included age and sex as covariates. For count outcomes, a negative binomial distribution with a log link and exchangeable covariance matrix was assumed. For dichotomous outcomes, a binomial distribution with a logit link and compound symmetry covariance was assumed. Patient-reported outcome measures were analyzed by standardized algorithms or scoring software. Analyses included established ADPKD disease progression risk factors such as baseline htTKV, age, gender, Mayo imaging classification,^5^ and CKD stage.

### Ethical Conduct

In accordance with the International Conference on Harmonization Good Clinical Practice Consolidated Guideline, and the applicable local laws and regulatory requirements of the countries in which the study was conducted, the study protocol was reviewed and approved by the governing institutional review board or independent ethics committee for each investigational site or country. Written informed consent was obtained from all subjects (or their guardian or legal representative, as applicable according to local laws). Subjects below the legal age of consent provided informed assent.

## Results

### Population Description

Out of 3479 subjects screened, 3409 were enrolled (422 in Asia Pacific, 1355 in Europe, 1373 in North America, and 259 in South America) (Supplementary Table S2; Supplementary Figures S1, S2). The mean (SD) follow-up was 589 (192) days with a median follow-up of 567 days. A majority, 2878 (84.4%), completed the month 12 visit and had both protocol-required MRIs, with 1139 (33.4%) completing the month 24 visit. Subjects represented a broad cross-section of patients with ADPKD across different ages (12 to 78 years), Mayo imaging classifications, and CKD stages (Table 1). The mean age of the subjects at baseline was lowest for those in CKD stage G1 (34.2 years) and increased with each CKD stage to 55.3 years for those in stage G5. At baseline, the majority of subjects were in CKD stage G1 to G3 (29.0%, 28.0%, and 26.4%, respectively), consistent with the original design of the study intended to enrich for earlier-stage patients. Overall, 2807 (82.3%) subjects had a family member with ADPKD, but the protocol did not include tracking or enrollment of affected family members.

**Table 1:** Baseline demographics and characteristics by CKD stage

Characteristic	All Enrolled^a^ (*N* = 3409)	Stage G1 (*n* = 990)	Stage G2 (*n* = 956)	Stage G3 (*n* = 899)	Stage G4 (*n* = 351)	Stage G5 (*n* = 101)
Age in yrs, mean (SD)	45.1 (12.9)	34.2 (10.8)	45.6 (10.7)	52.0 (9.8)	52.9 (9.0)	55.3 (9.7)
Female, *n* (%)	1891 (55.5)	620 (62.6)	515 (53.9)	476 (52.9)	168 (47.9)	55 (54.5)
Race, *n* (%)						
White	2918 (85.6)	851 (86.0)	816 (85.4)	783 (87.1)	302 (86.0)	75 (74.3)
Black or African-American	93 (2.7)	27 (2.7)	18 (1.9)	23 (2.6)	7 (2.0)	9 (8.9)
Asian	325 (9.5)	89 (9.0)	110 (11.5)	75 (8.3)	30 (8.5)	12 (11.9)
Other^b^	73 (2.1)	23 (2.3)	12 (1.3)	18 (2.0)	12 (3.4)	5 (5.0)
Hispanic or Latino ethnicity, *n* (%)	497 (14.6)	169 (17.1)	116 (12.1)	112 (12.5)	50 (14.2)	24 (23.8)
Height in cm, mean (SD)	171.0 (10.5)	170.8 (10.4)	171.8 (10.4)	170.8 (10.1)	170.8 (11.7)	168.8 (10.4)
Weight in kg, mean (SD)	78.0 (18.5)	73.8 (17.8)	78.5 (19.0)	80.4 (18.6)	81.8 (17.3)	78.6 (17.4)
Abdominal girth in cm, mean (SD)	93.2 (15.2)	87.9 (15.6)	92.8 (14.5)	96.3 (14.3)	98.9 (13.4)	99.0 (14.0)
Systolic BP in mm Hg, mean (SD)	130.2 (16.0)	126.0 (14.1)	130.3 (15.4)	132.0 (17.1)	135.3 (16.8)	135.6 (17.4)
Diastolic BP in mm Hg, mean (SD)	81.7 (10.7)	79.8 (10.4)	82.7 (10.6)	82.3 (10.9)	83.8 (10.6)	79.7 (10.2)
Family member with ADPKD, *n* (%)						
Yes	2807 (82.3)	839 (84.7)	790 (82.6)	733 (81.5)	284 (80.9)	72 (71.3)
No	367 (10.8)	93 (9.4)	107 (11.2)	96 (10.7)	38 (10.8)	20 (19.8)
Unknown	235 (6.9)	58 (5.9)	59 (6.2)	70 (7.8)	29 (8.3)	9 (8.9)
eGFR by CKD-EPI in ml/min per 1.73 m^2^, mean (SD)	69.3 (32.7)	108.2 (14.0)	75.5 (8.8)	45.7 (8.6)	23.1 (4.2)	10.0 (3.6)
TKV in ml, mean (SD)	1711.4 (1353.3)	930.6 (553.0)	1484.9 (957.5)	2184.4 (1431.1)	2897.9 (1717.5)	2920.4 (1691.9)
Height-adjusted TKV in ml/m, mean (SD)	994.8 (769.8)	544.2 (319.2)	860.1 (543.5)	1268.4 (810.8)	1681.9 (964.3)	1712.4 (936.7)
Mayo imaging classification, *n* (%)						
1A	203 (6.0)	78 (7.9)	66 (6.9)	43 (4.8)	9 (2.6)	0 (0.0)
1B	834 (24.5)	314 (31.7)	261 (27.3)	179 (19.9)	43 (12.3)	12 (11.9)
1C	1180 (34.6)	317 (32.0)	313 (32.7)	336 (37.4)	136 (38.7)	43 (42.6)
1D	680 (19.9)	162 (16.4)	187 (19.6)	196 (21.8)	95 (27.1)	25 (24.8)
1E	369 (10.8)	93 (9.4)	88 (9.2)	105 (11.7)	57 (16.2)	15 (14.9)
2	27 (0.8)	4 (0.4)	11 (1.2)	7 (0.8)	3 (0.9)	0 (0.0)
Type 1 switched to Type 2^c^	4 (0.1)	1 (0.1)	1 (0.1)	1 (0.1)	1 (0.3)	0 (0.0)
N/A^d^	112 (3.3)	21 (2.1)	29 (3.0)	32 (3.6)	7 (2.0)	6 (5.9)
ADPKD-related comorbidities, *n* (%)						
Hypertension	2303 (67.6)	462 (46.7)	648 (67.8)	738 (82.1)	295 (84.0)	90 (89.1)
Microalbuminuria	212 (6.2)	49 (4.9)	39 (4.1)	65 (7.2)	34 (9.7)	13 (12.9)
Proteinuria	802 (23.5)	172 (17.4)	184 (19.2)	246 (27.4)	130 (37.0)	39 (38.6)
Hematuria	999 (29.3)	242 (24.4)	252 (26.4)	299 (33.3)	127 (36.2)	40 (39.6)
Nephrolithiasis	596 (17.5)	131 (13.2)	164 (17.2)	180 (20.0)	75 (21.4)	26 (25.7)
Upper urinary tract infection	938 (27.5)	276 (27.9)	248 (25.9)	256 (28.5)	102 (29.1)	30 (29.7)
Anemia	534 (15.7)	93 (9.4)	101 (10.6)	158 (17.6)	102 (29.1)	54 (53.5)
Abdominal hernia	414 (12.1)	65 (6.6)	106 (11.1)	140 (15.6)	69 (19.7)	20 (19.8)
Colonic diverticula	158 (4.6)	18 (1.8)	43 (4.5)	51 (5.7)	30 (8.5)	10 (9.9)
Vascular/cardiac abnormalities	449 (13.2)	73 (7.4)	112 (11.7)	167 (18.6)	61 (17.4)	22 (21.8)
Nonhepato-renal cysts	431 (12.6)	101 (10.2)	110 (11.5)	138 (15.4)	55 (15.7)	13 (12.9)
Pharmacotherapies in use, *n* (%)						
RAAS inhibitors	1046 (30.7)	218 (22.0)	297 (31.1)	343 (38.2)	126 (35.9)	36 (35.6)
Analgesics	136 (4.0)	39 (3.9)	26 (2.7)	39 (4.3)	25 (7.1)	5 (5.0)
Antithrombotic agents	144 (4.2)	10 (1.0)	16 (1.7)	64 (7.1)	29 (8.3)	14 (13.9)
Beta-blocking agents	287 (8.4)	33 (3.3)	50 (5.2)	113 (12.6)	59 (16.8)	17 (16.8)
Calcium channel blockers	348 (10.2)	39 (3.9)	85 (8.9)	112 (12.5)	76 (21.7)	25 (24.8)
Diuretics	231 (6.8)	22 (2.2)	45 (4.7)	85 (9.5)	48 (13.7)	20 (19.8)
Lipid-modifying agents	389 (11.4)	41 (4.1)	67 (7.0)	160 (17.8)	84 (23.9)	18 (17.8)
Psychoanaleptics	109 (3.2)	21 (2.1)	29 (3.0)	36 (4.0)	12 (3.4)	7 (6.9)

ADPKD, autosomal dominant polycystic kidney disease; BP, blood pressure; CKD, chronic kidney disease; CKD-EPI, chronic kidney disease epidemiology collaboration formula; eGFR, estimated glomerular filtration rate; RAAS, renin-angiotensin-aldosterone system; TKV, total kidney volume.

Stage G1: eGFR ≥90 ml/min per 1.73 m^2^; Stage G2: eGFR ≥60 to <90 ml/min per 1.73 m^2^; Stage G3: eGFR ≥30 to <60 ml/min per 1.73 m^2^; Stage G4: eGFR ≥15 to <30 ml/min per 1.73 m^2^; Stage G5: eGFR <15 ml/min per 1.73 m^2^.

a For each characteristic, the sum of subjects in each CKD category is not the same as the number of subjects in the All Enrolled column. The study enrolled 19 subjects on dialysis and 4 kidney transplant recipients, who are included in the All Enrolled column but are not shown in CKD stage columns because of the very low subject numbers. Similarly, subjects who were missing CKD stage information at baseline are included in the All Enrolled column but not the columns for CKD category.

b Includes American Indian, Alaska Native, Native Hawaiian, Other Pacific Islander, and Other.

c Participants were reclassified from type 1 to type 2 following nephrectomy.

d The subject was either not within the Mayo imaging classification age range (<16 years or >80 years) or did not have a baseline height-adjusted TKV.

Baseline characteristics reflected disease progression, with worsening eGFR, TKV, abdominal girth, and greater frequency of comorbidities as CKD stage increased (Table 1). Use of pharmacotherapy for cardiovascular disease increased in later CKD stages, including but not limited to antithrombotics, inhibitors of the renin-angiotensin-aldosterone system, β-blockers, and calcium channel antagonists.

Lower proportions of subjects at later CKD stages had employment at baseline than those at earlier CKD stages (Table 2). Similarly, scores on measures of patient-reported quality of life were worse for subjects at later CKD stages, specifically, worse scores on the ADPKD-IS, SF-12v2 Physical Component Summary, and EQ-5D-3L index.

**Table 2:** Baseline employment status and patient-reported health-related quality of life by CKD stage

Characteristic	All enrolled^a^ (*N* = 3409)	Stage G1 (*n* = 990)	Stage G2 (*n* = 956)	Stage G3 (*n* = 899)	Stage G4 (*n* = 351)	Stage G5 (*n* = 101)
Employment status, *n* (%)						
Employed	2448 (71.8)	722 (72.9)	760 (79.5)	613 (68.2)	243 (69.2)	49 (48.5)
Full-time	2038 (59.8)	597 (60.3)	640 (66.9)	510 (56.7)	200 (57.0)	40 (39.6)
Part-time	377 (11.1)	120 (12.1)	114 (11.9)	94 (10.5)	35 (10.0)	5 (5.0)
Part-time due to ADPKD	33 (1.0)	5 (0.5)	6 (0.6)	9 (1.0)	8 (2.3)	4 (4.0)
Not Employed	960 (28.2)	268 (27.1)	196 (20.5)	286 (31.8)	108 (30.8)	52 (51.5)
Retired	279 (8.2)	10 (1.0)	56 (5.9)	123 (13.7)	60 (17.1)	19 (18.8)
Retired due to ADPKD	49 (1.4)	4 (0.4)	6 (0.6)	17 (1.9)	7 (2.0)	7 (6.9)
Homemaker	241 (7.1)	65 (6.6)	70 (7.3)	66 (7.3)	18 (5.1)	10 (9.9)
Unable to find work	57 (1.7)	19 (1.9)	19 (2.0)	10 (1.1)	5 (1.4)	3 (3.0)
Unable to work due to health	63 (1.8)	14 (1.4)	10 (1.0)	27 (3.0)	6 (1.7)	4 (4.0)
Unable to work due to ADPKD	57 (1.7)	7 (0.7)	8 (0.8)	18 (2.0)	10 (2.8)	6 (5.9)
Student	150 (4.4)	127 (12.8)	15 (1.6)	2 (0.2)	0 (0.0)	0 (0.0)
Other	64 (1.9)	22 (2.2)	12 (1.3)	23 (2.6)	2 (0.6)	3 (3.0)
ADPKD-IS scores^b^ (*n*)	1108	266	299	337	127	37
Physical, mean (SD)	1.59 (0.85)	1.47 (0.82)	1.44 (0.73)	1.61 (0.84)	1.83 (0.88)	2.33 (1.05)
Emotional, mean (SD)	1.78 (0.88)	1.72 (0.88)	1.66 (0.83)	1.76 (0.86)	2.07 (0.91)	2.27 (0.95)
Fatigue, mean (SD)	1.90 (1.08)	1.77 (1.03)	1.71 (0.97)	1.91 (1.05)	2.25 (1.23)	2.56 (1.18)
ADPKD-UIS scores^b^ (*n*)	1108	266	299	337	127	37
Frequency, mean (SD)	1.43 (0.74)	1.45 (0.84)	1.36 (0.63)	1.46 (0.77)	1.48 (0.74)	1.53 (0.76)
Urgency, mean (SD)	1.39 (0.73)	1.39 (0.82)	1.34 (0.65)	1.42 (0.75)	1.43 (0.71)	1.41 (0.76)
Nocturia, mean (SD)	1.85 (1.00)	1.71 (1.05)	1.71 (0.88)	1.94 (0.96)	2.24 (1.17)	1.83 (0.93)
BPI-SF scores (*n*)	2480	727	702	660	254	72
Composite pain severity,^c^ mean (SD)	0.98 (1.53)	0.91 (1.47)	0.90 (1.40)	1.03 (1.60)	1.07 (1.54)	1.46 (2.11)
SF-12v2 scores^d^ (*n*)	1931	512	575	546	196	49
PCS, mean (SD)	49.94 (9.69)	52.22 (8.41)	51.22 (8.56)	48.65 (10.44)	46.79 (9.89)	40.74 (11.46)
MCS, mean (SD)	50.15 (9.61)	49.66 (9.90)	50.30 (9.34)	50.44 (9.37)	51.09 (9.63)	47.59 (11.60)
EQ-5D-3L index US^e^ (*n*)	1889	558	550	496	193	46
Mean (SD)	0.91 (0.13)	0.92 (0.12)	0.92 (0.13)	0.90 (0.14)	0.89 (0.14)	0.83 (0.15)

ADPKD, autosomal dominant polycystic kidney disease; ADPKD-IS, Autosomal Dominant Polycystic Kidney Disease-Impact Scale; ADPKD-UIS, Autosomal Dominant Polycystic Kidney Disease-Urinary Impact Scale; BPI-SF, Brief Pain Inventory-Short Form; CKD, chronic kidney disease; MCS, Mental Component Summary; PCS, Physical Component Summary; SF-12v2, Short Form 12-item Health Survey version 2; US, United States.

Stage G1: eGFR ≥90 ml/min per 1.73 m^2^; Stage G2: eGFR ≥60 to <90 ml/min per 1.73 m^2^; Stage G3: eGFR ≥30 to <60 ml/min per 1.73 m^2^; Stage G4: eGFR ≥15 to <30 ml/min per 1.73 m^2^; Stage G5: eGFR <15 ml/min per 1.73 m^2^.

a For each characteristic, the sum of subjects in each CKD category is not the same as the number of subjects in the All Enrolled column. The study enrolled 19 subjects on dialysis and 4 kidney transplant recipients, who are included in the All Enrolled column but are not shown in CKD stage columns because of the very low subject numbers. Similarly, subjects who were missing CKD stage information at baseline are included in the All Enrolled column but not the columns for CKD category.

b Each domain is scored on a range of 1–5, with 1 indicating “not difficult at all” or “not bothered at all” and 5 indicating “extremely difficult” or “extremely bothered.”

c The Composite Pain Severity score ranges from 0 (least severe) to 10 (most severe) and is a mean of scores for worst, least, average, and current pain.

d PCS and MCS each range from 0 (worst) to 100 (best).

e Scores for the 5 dimensions of mobility, self-care, usual activities, pain or discomfort, and anxiety or depression were converted into a summary index using reference norms for the US population. Higher scores indicate better quality of life.

**Figure 1:**
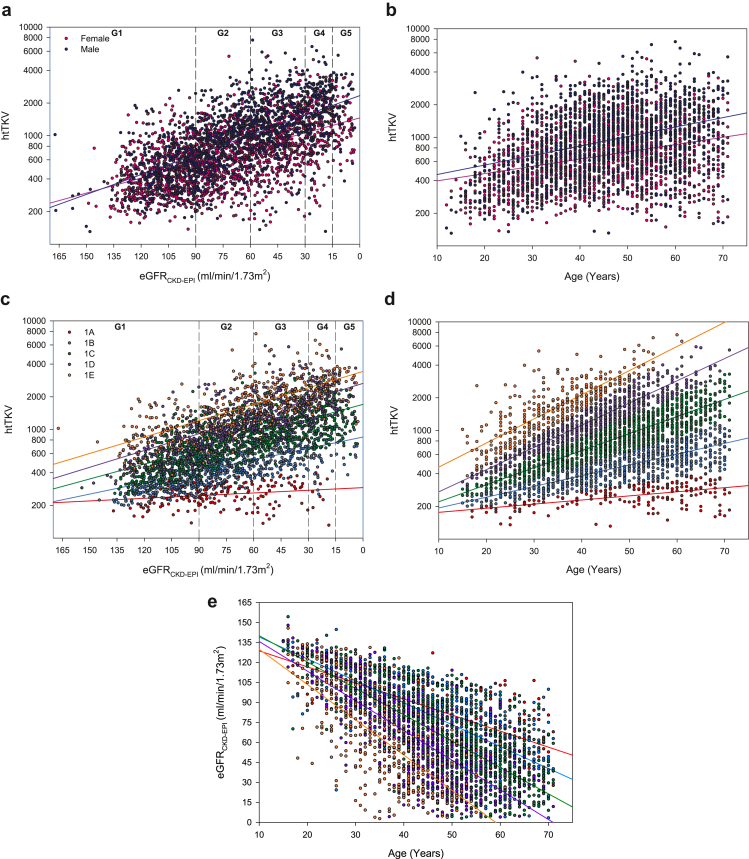
(a) Baseline htTKV and eGFR by sex; (b) baseline htTKV and age by sex; (c) baseline htTKV and eGFR by Mayo imaging classification; (d) baseline htTKV and age by Mayo imaging classification; (e) baseline eGFR and age by Mayo imaging classification. CKD-EPI, Chronic Kidney Disease Epidemiology Collaboration Formula; eGFR, estimated glomerular filtration rate; G1–G5, chronic kidney disease stages 1–5; htTKV, height-adjusted total kidney volume.

Baseline relationships among htTKV, eGFR, and age by sex and Mayo imaging classification are shown in Figure 1 and Table 3. Men had higher htTKV than women in each CKD stage. Subjects in Mayo imaging subclass 1A had mean htTKV ranging from 241.4 ml/m to 271.7 ml/m across CKD stages, and subjects in subclass 1E had mean htTKV ranging from 957.4 ml/m to 2863.6 ml/m across CKD stages. Given that Mayo risk classification is based on htTKV adjusted for age, subjects were younger in each progressive Mayo risk subclass by CKD stage (subclass 1A: mean age of 40.3 years to 60.2 years across CKD stages; versus subclass 1E: 25.5 years to 42.4 years across CKD stages).

**Table 3:** Baseline ADPKD risk characteristics by CKD stage

Characteristic	Stage G1 *n* = 990	Stage G2 *n* = 956	Stage G3 *n* = 899	Stage G4 *n* = 351	Stage G5 *n* = 101
By sex					
Females, *n* (%)	620 (62.6)	515 (53.9)	476 (52.9)	168 (47.9)	55 (54.5)
Mean age (SD), yrs	34.6 (10.8)	46.0 (10.7)	52.2 (9.2)	52.9 (8.4)	54.3 (8.7)
Mean htTKV (SD), ml/m	522.8 (300.1)	757.0 (469.5)	1057.2 (622.3)	1345.9 (737.2)	1451.1 (722.8)
Males, *n* (%)	370 (37.4)	441 (46.1)	423 (47.1)	183 (52.1)	46 (45.5)
Mean age (SD), yrs	33.6 (10.9)	45.1 (10.9)	51.8 (10.5)	53.0 (9.6)	55.6 (9.9)
Mean htTKV (SD), ml/m	579.8 (346.1)	978.4 (597.9)	1506.1 (925.9)	1990.4 (1041.3)	2049.0 (1072.0)
By Mayo imaging classification					
Mean age (SD) in yrs, *n*					
1A	40.3 (14.0), 78	55.4 (9.9), 66	60.2 (6.9), 43	56.2 (10.7), 9	
1B	37.4 (10.7), 314	49.7 (10.2), 261	58.7 (7.0), 179	59.1 (8.4), 43	61.2 (5.0), 12
1C	34.4 (9.5), 317	46.2 (9.2), 313	54.3 (7.5), 336	56.6 (7.3), 136	58.8 (6.8), 43
1D	30.8 (8.5), 162	40.7 (7.5), 187	47.3 (7.7), 196	51.1 (6.5), 95	53.7 (7.9), 25
1E	25.5 (6.2), 93	33.1 (7.5), 88	38.5 (7.4), 105	42.4 (6.4), 57	39.9 (6.3), 15
2	41.8 (16.1), 4	53.3 (13.8), 11	52.9 (7.2), 7	50.0 (3.6), 3	
Type 1 switched to Type 2^a^	16.0 (N/A), 1	50.0 (N/A), 1	59.0 (N/A), 1	60.0 (N/A), 1	
Mean htTKV (SD) in ml/m, *n*					
1A	241.4 (50.3), 78	268.3 (49.5), 66	269.5 (68.6), 43	271.7 (84.3), 9	
1B	370.9 (111.7), 314	497.1 (150.4), 261	621.2 (167.7), 179	659.5 (211.7), 43	688.1 (184.4), 12
1C	558.4 (235.4), 317	854.3 (307.6), 313	1166.6 (414.8), 336	1345.6 (519.9), 136	1343.8 (444.6), 43
1D	764.5 (346.0), 162	1224.5 (496.9), 187	1743.9 (822.4), 196	2070.5 (657.5), 95	2335.6 (1013.6), 25
1E	957.4 (415.5), 93	1645.2 (782.1), 88	2236.4 (1021.4), 105	2863.6 (1086.4), 57	2550.0 (865.4), 15
2	950.7 (502.9), 4	735.3 (453.8), 11	943.5 (433.2), 7	1073.5 (506.2), 3	
Type 1 switched to Type 2^a^	765.7 (N/A), 1	625.0 (N/A), 1	1729.9 (N/A), 1	1632.1 (N/A), 1	

ADPKD, autosomal dominant polycystic kidney disease; CKD, chronic kidney disease; htTKV, height-adjusted total kidney volume.

a Participants were reclassified from type 1 to type 2 following nephrectomy.

### Baseline Characteristics and Outcomes

**Figure 2:**
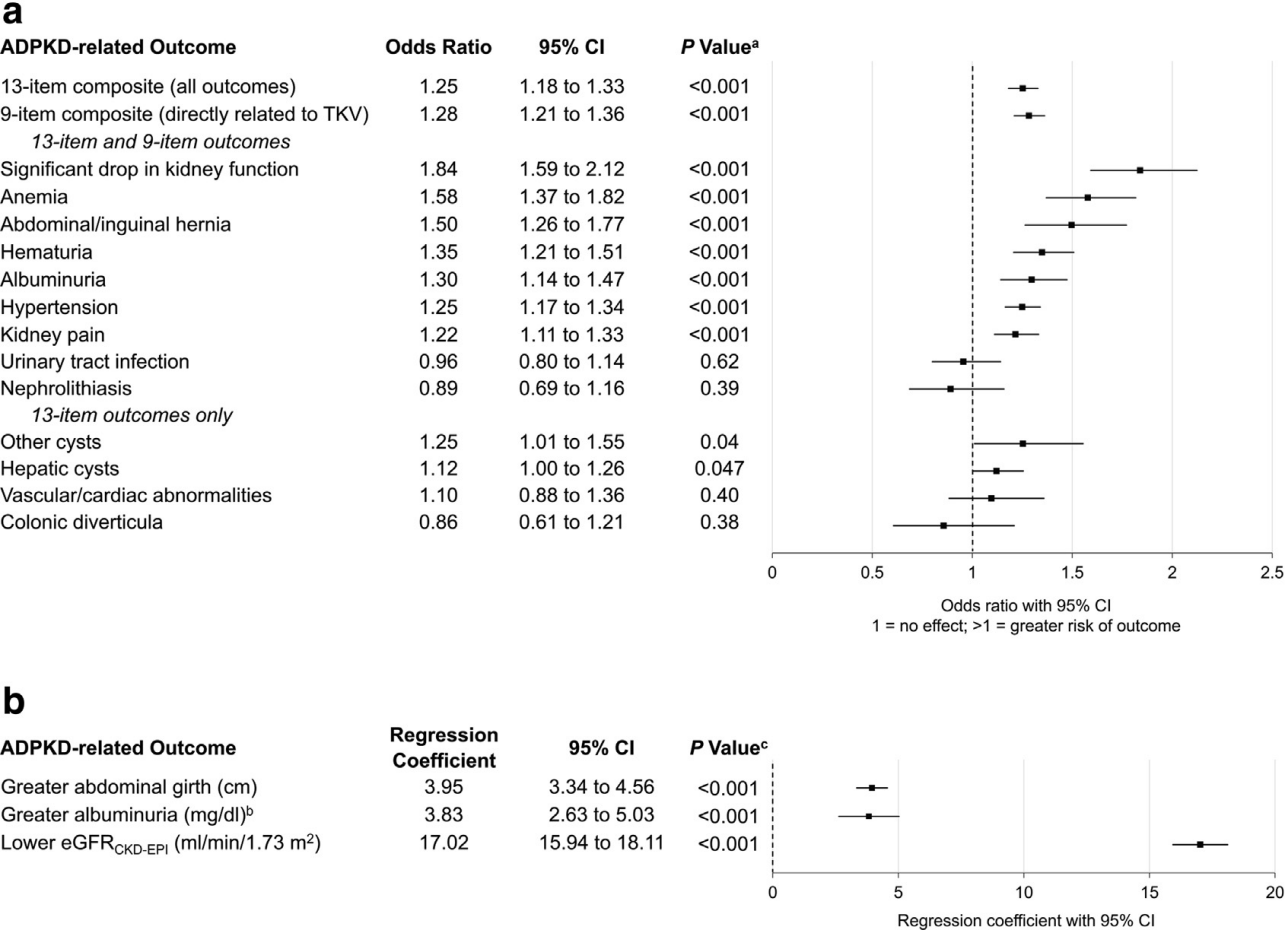
Association of each l/m in baseline htTKV with adverse (a) binary and (b) continuous ADPKD-related outcomes (enrolled population). ^a^*P*-values derived from generalized estimating equation marginal models with repeated measurement in which there are subject, sex, and visit as groups, and age and baseline htTKV as covariates. ^b^Albumin/creatinine ratio. ^c^*P*-values derived from mixed models with repeated measurement in which there are subject, sex, and visit as groups, and age and baseline htTKV as covariates. ADPKD, autosomal dominant polycystic kidney disease; CI, confidence interval; CKD-EPI, Chronic Kidney Disease Epidemiology Collaboration; eGFR, estimated glomerular filtration rate; htTKV, height-adjusted total kidney volume; TKV, total kidney volume.

**Figure 3:**
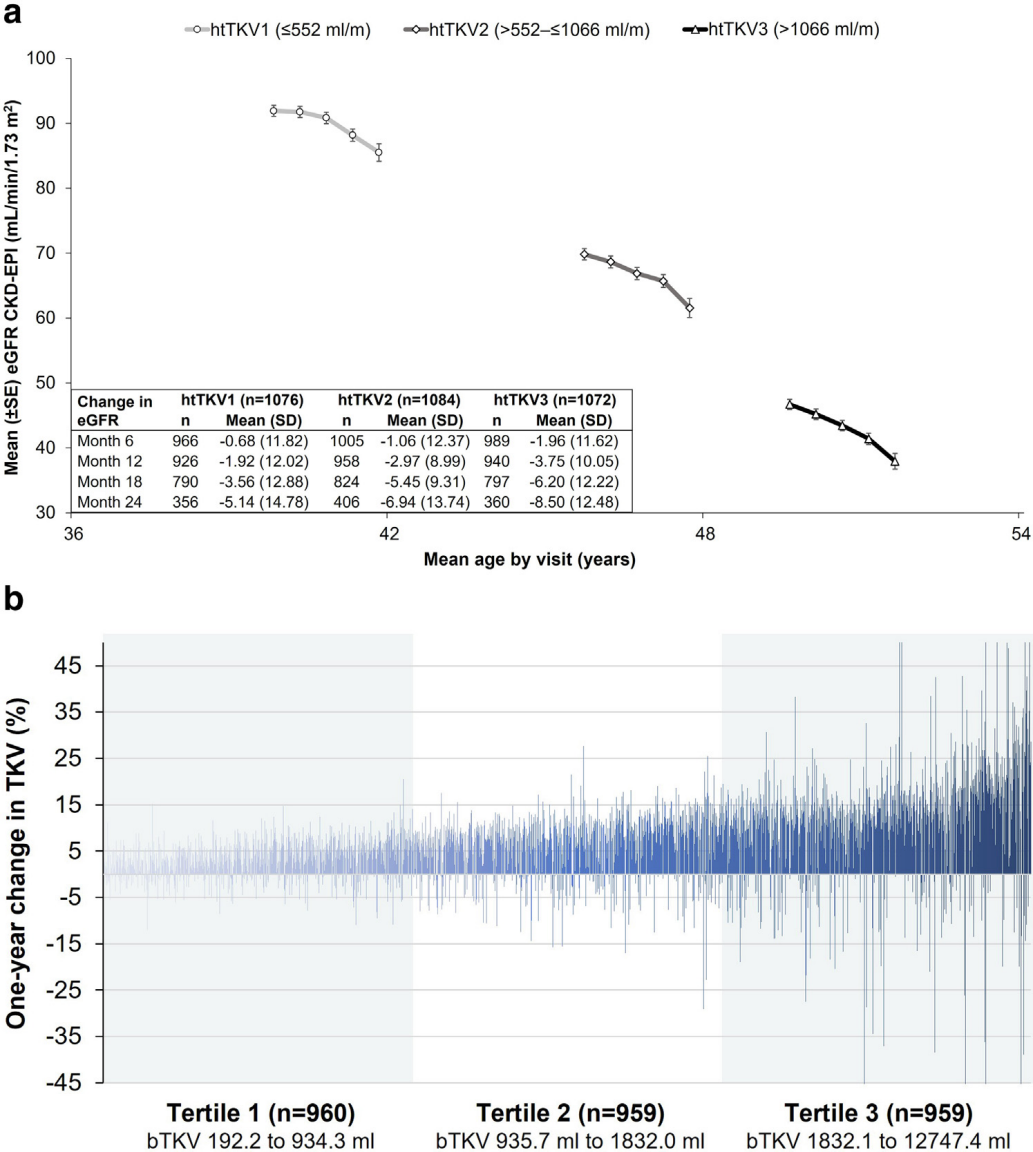
Changes in kidney function and total kidney volume. (a) eGFR over time (at baseline and subsequent 6-month intervals) by baseline htTKV tertile. (b) Percent change in total kidney volume at Month 12 visit by htTKV tertile. In Figure 3b, individual subjects’ percent change in TKV at the 12-month visit are plotted in ascending order by baseline TKV from left (smallest baseline TKV) to right (largest baseline TKV). Nine of 2878 subjects’ bars go beyond the scale of the graph: 3 subjects with declines of 52.3, 56.5, and 72.0% and 6 subjects with increases of 52.9, 53.0, 57.3, 61.2, 62.8, and 76.2% during the observation period. bTKV, baseline total kidney volume; CKD-EPI, Chronic Kidney Disease Epidemiology Collaboration; eGFR, estimated glomerular filtration rate; htTKV, height-adjusted total kidney volume; SD, standard deviation; SE, standard error; TKV, total kidney volume.

Baseline htTKV (each 1 l/m increase) was associated with the occurrence of most of the ADPKD-related clinical outcomes assessed (Figure 2a) individually and when combined in a 13-item and a 9-item (those related to TKV) composite. Baseline htTKV was also associated with worse continuous clinical outcomes (Figure 2b), namely abdominal girth, albumin-to-creatinine ratio, and kidney function (eGFR_CKD-Epidemiology Collaboration_). In an analysis by baseline htTKV tertile, subjects in the greatest baseline htTKV tertile were older and had lower eGFR at baseline, exhibited more rapid eGFR decline over time (Figure 3a), and exhibited greater eGFR decline at 24 months (Supplementary Figure S3A). Similarly, greater proportions of subjects with more severe Mayo risk classifications experienced eGFR decline of 30% or greater at 24 months than those with less severe risk classifications (Supplementary Figure S3B). Given that a more severe Mayo risk classification is associated with more rapid progression, few older subjects were in risk subclasses 1D or 1E (Supplementary Figure S4).

**Table:** 

Number of Participants Contributing Covariates and Outcomes by Time Point	
Figure 2a	Figure 2b
Baseline: *N* = 3409; *n* = 3305 (96.9%) Month 6: *N* = 3076; *n* = 3063 (99.6%) Month 12: *N* = 2943; *n* = 2934 (99.7%) Month 18: *N* = 2514; *n* = 2508 (99.8%) Month 24: *N* = 1162; *n* = 1161 (99.9%) Month 30: *N* = 209; *n* = 209 (100%) Month 36: *N* = 5; *n* = 5 (100%)	Baseline: *N* = 3319; *n* = 3232 (97.4%) Month 6: *N* = 3023; *n* = 3013 (99.7%) Month 12: *N* = 2874; *n* = 2868 (99.8%) Month 18: *N* = 2453; *n* = 2447 (99.8%) Month 24: *N* = 1139; *n* = 1138 (99.9%) Month 30: *N* = 216; *n* = 216 (100%) Month 36: *N* = 6; *n* = 6 (100%)

**Figure 4:**
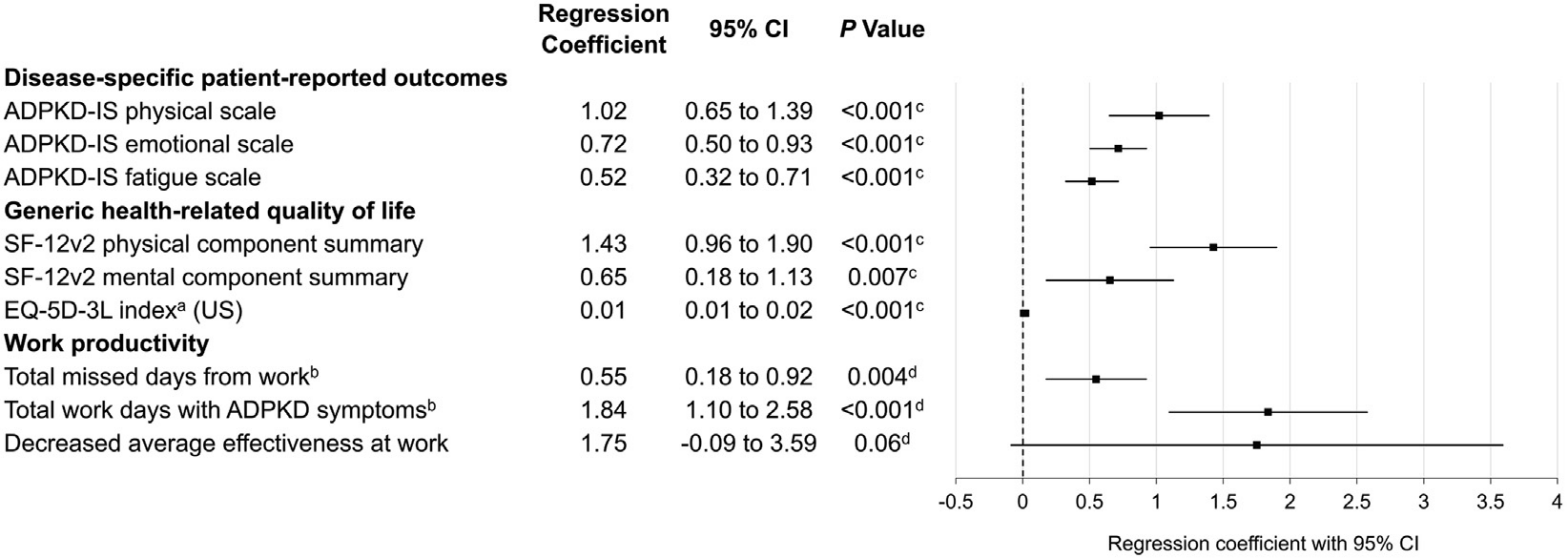
Associations of each l/m in baseline height-adjusted total kidney volume with worse patient-reported outcomes. ^a^Scored using reference norms for the US population. ^b^For the duration of the study. ^c^*P*-value derived from mixed models with repeated measurement in which there are subject, sex and visit as groups, and age and baseline htTKV as covariates. ^d^*P*-value derived from analysis of covariance models in which sex as group, and age and baseline htTKV as covariates. For the ADPKD-IS, SF-12v2, and EQ-5D-3L index, the associations of greater htTKV with worse quality of life were expressed as higher scores on the ADPKD-IS scales and lower scores on the SF-12v2 components and EQ-5D-3L index. ADPKD, autosomal dominant polycystic kidney disease; ADPKD-IS, Autosomal Dominant Polycystic Kidney Disease Impact Scale; CI, confidence interval; htTKV, height-adjusted total kidney volume; SF-12v2, Short Form 12-item Health Survey version 2; US, United States.

Baseline htTKV was associated with worse scores on multiple measures of patient-reported quality of life, including the ADPKD-IS, SF-12v2, EQ-5D-3L index, and work productivity parameters (more missed days, days working with ADPKD symptoms, or decreased effectiveness while working) (Figure 4). For the ADPKD-IS, SF-12v2, and EQ-5D-3L index, the association with greater htTKV was tied to disease-specific measures or measures of physical functioning.

**Table:** 

Number of Participants Contributing Outcome by Time Point			
ADPKD-IS	SF-12v2	EQ-5D-3L	Work productivity
Baseline: *n* = 1108 Month 6: *n* = 1013 Month 12: *n* = 1038 Month 18: *n* = 867 Month 24: *n* = 480 Month 30: *n* = 106 Month 36: *n* =3	Baseline: *n* = 1931 Month 6: *n* = 1822 Month 12: *n* = 1701 Month 18: *n* = 1409 Month 24: *n* = 746 Month 30: *n* = 149 Month 36: *n* = 3	Baseline: *n* = 1889 Month 6: *n* = 2048 Month 12: *n* = 1996 Month 18: *n* = 1717 Month 24: *n* = 894 Month 30: *n* = 170 Month 36: *n* = 3	Baseline: *n* = 3409 Month 6: *n* = 3076 Month 12: *n* = 2943 Month 18: *n* = 2514 Month 24: *n* = 1162 Month 30: *n* = 209 Month 36: *n* = 5

**Figure 5:**
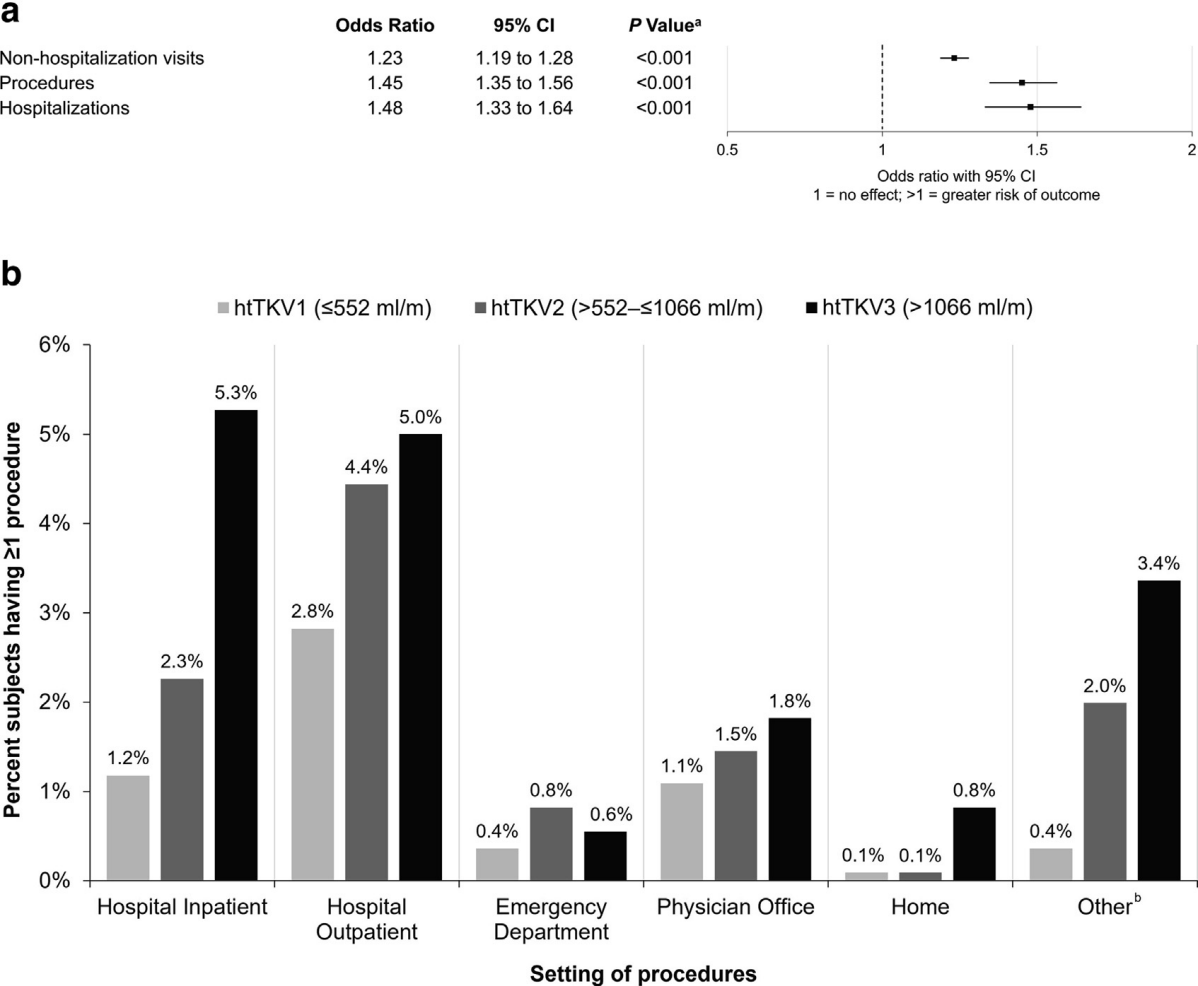
Impact of htTKV on health care resource utilization in ADPKD. (a) Association of each l/m in baseline htTKV with health care resource utilization. (b) Percentage of subjects having at least one procedure by htTKV tertile. ^a^*P*-values derived from generalized linear models with Poisson distribution in which there are subject, sex as groups, and age and baseline htTKV as covariates. ^b^ For example, ultrasound, MRI, X-ray, hemodialysis. ADPKD, autosomal dominant polycystic kidney disease; CI, confidence interval; htTKV, height-adjusted total kidney volume.

Baseline htTKV was also positively associated with subsequent self-reported health care resource utilization during the study follow-up period (nonhospitalization visits, procedures, and hospitalizations) (Figure 5a). In an analysis by baseline htTKV tertile, subjects with larger htTKV more frequently underwent medical procedures during study follow-up (Figure 5b). A total of 737 procedures were reported by 308 (9.0%) subjects during follow-up. The mean (SD) number of procedures reported in those affected was 2.7 (20.9) per year. Sixty-seven percent of all procedures were performed at either hospital outpatient (150 subjects, 315 procedures) or inpatient (99 subjects, 177 procedures) locations. Types of health insurance in the study population are shown in Supplementary Table S3.

Of the 3409 subjects, 1065 (31.2%) reported utilizing hospitalization (245 [7.2%]) and nonhospitalization (990 [29.0%]) medical resources at least once during the study, with a mean (SD) of 2.2 (2.9) visits per year, in addition to care provided as part of study visits. The highest percentage of subjects reported physician’s office visits (18.8%), followed by hospital outpatient visits (10.0%), and emergency department visits (4.2%). Subjects with worse CKD at baseline had a greater mean (SD) number of nonhospitalization health care visits per year as follows: Stage G1, 1.7 (1.7); G2, 2.1 (2.9); G3, 2.1 (2.2); G4, 2.4 (3.3); and G5, 4.5 (5.6).

### Rates of ADPKD Progression During Follow-up

Among the 2872 subjects without a history of kidney transplant, the mean (SD) TKV growth rate was 6.2% (7.9) from baseline to the month 12 visit. Some subjects had large changes in TKV ranging from a 72.7% decrease to a 60.4% increase. Though rare, large changes in TKV were typically seen in subjects with larger kidneys at baseline (Figure 3b, percent change in TKV; Supplementary Figure S5A, absolute change in TKV). Only 41 subjects had a 30% or greater change (either increase or decrease) in TKV. Approximately 85% of subjects had an increase in TKV during the follow-up period, with some subjects experiencing a decline in TKV (Supplementary Figure S5B).

The mean baseline eGFR by baseline htTKV tertile was 91.9 ml/min per 1.73 m^2^ for tertile 1 (≤552 ml/m), 69.8 ml/min per 1.73 m^2^ for tertile 2 (>552 to ≤1066 ml/m), and 46.7 ml/min per 1.73 m^2^ for tertile 3 (>1066 ml/m). The mean (SD) decline in eGFR at 1 year was −1.9 (12.0) ml/min per 1.73 m^2^ for tertile 1, −3.0 (9.0) ml/min per 1.73 m^2^ for tertile 2, and −3.8 (10.1) ml/min per 1.73 m^2^ for tertile 3 (Figure 3a).

The mean baseline abdominal girth by baseline htTKV tertile was 88.0 cm for tertile 1, 92.3 cm for tertile 2, and 98.6 cm for tertile 3. The mean (SD) increase in abdominal girth at 1 year was 0.1 (8.9) cm for tertile 1, 0.6 (7.8) cm for tertile 2, and 0.7 (6.9) cm for tertile 3.

The proportion of subjects who had employment declined slightly over time among subjects who were in later stages of CKD at baseline, although changes were small in most CKD categories (baseline vs. Month 12) as follows: G1 (72.9 vs. 74.4%), G2 (79.5 vs. 79.3%), G3 (68.2 vs. 67.0%), G4 (69.2 vs. 64.0%), and G5 (48.5 vs. 39.5%).

## Discussion

OVERTURE provides longitudinal data on ADPKD progression from the largest prospective global cohort studied till date. The study included a broad cross-section of subjects at different disease stages and results support the value of baseline htTKV at a given age for predicting kidney function decline in ADPKD. Baseline htTKV was also strongly predictive of the occurrence of ADPKD-related outcomes such as kidney pain, hematuria, albuminuria, anemia, hypertension, and abdominal or inguinal hernia. The relationship of htTKV at baseline with worse disease-related outcomes was reflected in higher health care resource utilization and worse patient-reported quality of life on multiple measures during follow-up.

The associations between TKV-related variables and ADPKD outcomes identified in OVERTURE are consistent with results from the CRISP cohort, showing that baseline TKV is associated with loss of kidney function in ADPKD.^4^^,^^7^^,^^15^ The OVERTURE data extend the CRISP findings from a young population with relatively preserved kidney function to a global cross-section of patients with ADPKD at different ages and stages of CKD. The results further support the importance of incorporating factors such as age and cyst burden when estimating the risk of disease progression. It should be noted, however, that the relationship of htTKV to eGFR decline was attenuated in subjects at lesser risk of rapid progression by Mayo imaging classification (i.e, subclasses 1A–1B). Plots of baseline data showed that htTKV remained relatively low in the lesser Mayo risk classifications, even as eGFR declined with age in all risk classifications. This finding is consistent with recent data from the Mayo Clinic that htTKV at time of kidney failure exhibits an inverse correlation with patient age at kidney failure.^27^ Whereas cystic expansion appears to be the predominant factor in declining kidney function among rapidly progressing patients, other factors, such as vascular disease and interstitial fibrosis, may play a greater role in impairing kidney function in patients who progress slowly. Conversely, large percentage drops in eGFR were observed in a small proportion of subjects in the lowest htTKV tertile at baseline (Supplementary Figure S3a), underscoring the limitations of kidney volume alone in estimating risk of progression.

OVERTURE also builds on previous research that found ADPKD affects health care resource utilization and work productivity. One study of privately insured PKD patients in the United States showed that medical and pharmacy costs correlated inversely with kidney function.^18^ Other data indicated that health care resource utilization in patients with ADPKD is greater than in age-matched and sex-matched healthy controls, presumably due to pain and treatment for high blood pressure, bladder and kidney infections, hematuria, and cyst-related complications.^19^ In OVERTURE, greater baseline htTKV was associated with higher subsequent health resource utilization (nonhospitalization visits, procedures, and hospitalizations) and with longitudinal decreases in parameters of patient-reported work productivity. Employment status was stable during the observation period, which may be attributable to the 12-month time period for follow-up of this parameter and the fact that decreased employment among subjects at more advanced CKD stages was already evident at baseline.

The data supports the point that clinical trial populations (e.g., TEMPO 3:4, REPRISE, HALT), while enriched for patients likely to progress rapidly, nonetheless collectively represented a broad spectrum of ADPKD stages, mapping well to the OVERTURE population.

A limitation of this study was the relatively short follow-up (1-year for TKV, up to 3 years for other outcomes), because ADPKD typically progresses over decades. In addition, the study population was largely in earlier stages of CKD, so event rates and overall disease progression were more limited for some study outcomes. This is likely to impact the ability to accurately measure changes in less common ADPKD progression events and limited further analyses of some data, such as health care resource utilization over time. Neveretheless, with the large overall number of participants across the continuum of ADPKD progression, even relatively rare events were detected during the study. Another limitation is that the study sites, though distributed internationally, enrolled a population with a predominantly European ancestry and with only 3% Black or African-American participants. More data on the possible effects of race and ethnicity on the relationship of htTKV with ADPKD outcomes are needed. The study design is observational, with many variables provided as available in medical records without independent verification by a central laboratory (with the exception of TKV, albumin-to-creatinine ratio, urine osmolality, and serum creatinine). The prospective collection of such information in real time, however, is likely to enhance its accuracy. Fewer than 1% of participants were found to have class 2 Mayo imaging classification, which is lower compared to previous findings,^28^ suggesting a need for greater standardization of MRI protocols. Analysis approaches such as age-adjusting TKV may provide additional insights to predict disease progression. In addition, self-report is the preferred method for collection of certain types of outcomes, such as quality of life or disease-specific life impacts that were captured in patient-reported outcome questionnaires. Genetic testing was not performed as part of the study and only reported as a diagnostic factor for 4.3% of participants.

Studies such as this are important to gain a broader understanding of the factors affecting ADPKD disease progression, including impacts on patient-reported quality of life, health care resource utilization, and productivity. Findings from the OVERTURE study provide an important opening to future research in real-world ADPKD populations across a variety of potential outcomes.

## Disclosure

RDP received research funding from Otsuka, Kadmon Corporation, Palladio Bio, Sanofi-Genzyme, and Reata; has been a consultant to Palladio Bio, Reata, Sanofi-Genzyme, Navitor, Carraway, and Otsuka and is section editor for Renal Cystic Diseases, UpToDate. DO is an employee of Otsuka. JO is a former employee of Otsuka. DGB was a paid consultant to and Speaker Bureau member for Otsuka Canada; and their clinical research unit receives grants from Otsuka Canada. KB received research funds and/or honoraria from AbbVie, Alexion, Astellas, Bristol-Myers Squibb, Chiesi, Fresenius, Hansa, Hexal, Novartis, Otsuka, Pfizer, Roche, Sandoz, Stada, Veloxis, and Vifor. ABC is a consultant for Otsuka. SH declares the following from Otsuka: advisor, consulting, scientific grant, honorarium. ACMO declares consultancy agreements from the following: Galapagos, Mironid, ONO, Palladio, and Sanofi-Genzyme; unrestricted educational grant from Otsuka Europe; and that all money is paid to his employing institution. VET declares consultancy agreements from Otsuka Pharmaceuticals, Sanofi, Palladio, Blueprint Medicines, Mironid, Reata, Regulus; research funding from Otsuka Pharmaceuticals, Palladio Biosciences, Mironid, Sanofi-Genzyme, Blueprint Medicines, Reata, Regulus (all preclinical trial, preclinical research or clinical trials); honoraria from Otsuka Pharmaceuticals [to institution]; and scientific advisor or membership of Palladio, Otsuka Pharmaceuticals, Mironid, Reata, and Sanofi-Genzyme. ANT declares being on the Advisory Board and a Speaker at Otsuka from 2012 to 2018; funds were paid to institution. HK declares being an employee of Otsuka from 2001 to 2019 and being consultant for Otsuka 2019 to 2021. BYG has no conflict of interest to declare.

## Supplementary Material

Supplementary File (PDF)

**Figure S1.** Number of participants per site.

**Figure S2.** Subject flow.

**Figure S3.** Percentage of subjects reaching eGFR decline thresholds at 24 months (a) by baseline htTKV tertile and (b) by baseline Mayo imaging classification.

**Figure S4.** Distribution of Mayo imaging classification by age group.

**Figure S5.** One-year change in TKV. (a) Absolute change in TKV at Month 12 visit – individual subjects in ascending order by baseline TKV. (b) Subjects grouped by percent change in TKV at Month 12 visit.

**Table S1.** Study enrollment criteria.

**Table S2.** Number of participants by region and country.

**Table S3.** Types of health insurance in the study population.

**List of Principal Investigators and Study Sites.**

**STROBE Statement.**
