# Supplementary material for: OVERTURE: A Worldwide, Prospective, Observational Study of Disease Characteristics in Patients With ADPKD
Source: Kidney Int Rep. 2023 Feb 13;8(5):989–1001. doi: 10.1016/j.ekir.2023.02.1073 (PMC10166786; doi:10.1016/j.ekir.2023.02.1073)
Supplement: Supplementary File (PDF) [file mmc1.pdf]

SUPPLEMENTARY MATERIAL

STROBE Statement

	Item No	Recommendation	Page No
**Title and abstract**	1	(*a*) Indicate the study's design with a commonly used term in the title or the abstract	1, 3
		(*b*) Provide in the abstract an informative and balanced summary of what was done and what was found	3
**Introduction**			
Background/rationale	2	Explain the scientific background and rationale for the investigation being reported	5-6
Objectives	3	State specific objectives, including any prespecified hypotheses	6, 9
**Methods**			
Study design	4	Present key elements of study design early in the paper	6-10
Setting	5	Describe the setting, locations, and relevant dates, including periods of recruitment, exposure, follow-up, and data collection	7, 10-11
Participants	6	(*a*) Give the eligibility criteria, and the sources and methods of selection of participants. Describe methods of follow-up	7-9
		(*b*) For matched studies, give matching criteria and number of exposed and unexposed	n/a
Variables	7	Clearly define all outcomes, exposures, predictors, potential confounders, and effect modifiers. Give diagnostic criteria, if applicable	7-9
Data sources/ measurement	8*	For each variable of interest, give sources of data and details of methods of assessment (measurement). Describe comparability of assessment methods if there is more than one group	7-9
Bias	9	Describe any efforts to address potential sources of bias	16
Study size	10	Explain how the study size was arrived at	9
Quantitative variables	11	Explain how quantitative variables were handled in the analyses. If applicable, describe which groupings were chosen and why	10
Statistical methods	12	(*a*) Describe all statistical methods, including those used to control for confounding	10
		(*b*) Describe any methods used to examine subgroups and interactions	10
		(*c*) Explain how missing data were addressed	Tables 1, 2
		(*d*) If applicable, explain how loss to follow-up was addressed	Figures 2, 4
		(*e*) Describe any sensitivity analyses	n/a
**Results**			
Participants	13*	(a) Report numbers of individuals at each stage of study—eg numbers potentially eligible, examined for eligibility, confirmed eligible, included in the study, completing follow-up, and analysed	Figure S2

		(b) Give reasons for non-participation at each stage	Figure S2
		(c) Consider use of a flow diagram	Figure S2
Descriptive data	14*	(a) Give characteristics of study participants (eg demographic, clinical, social) and information on exposures and potential confounders	Tables 1, 2
		(b) Indicate number of participants with missing data for each variable of interest	Figures 2, 4
		(c) Summarise follow-up time (eg, average and total amount)	10-11
Outcome data	15	Report numbers of outcome events or summary measures over time	12-14

Main results	16	(*a*) Give unadjusted estimates and, if applicable, confounder-adjusted estimates and their precision (eg, 95% confidence interval). Make clear which confounders were adjusted for and why they were included	10, Figures 2-5
		(*b*) Report category boundaries when continuous variables were categorized	Figures S3, S5b
		(*c*) If relevant, consider translating estimates of relative risk into absolute risk for a meaningful time period	n/a
Other analyses	17	Report other analyses done—eg analyses of subgroups and interactions, and sensitivity analyses	11-14
**Discussion**			
Key results	18	Summarise key results with reference to study objectives	14-17
Limitations	19	Discuss limitations of the study, taking into account sources of potential bias or imprecision. Discuss both direction and magnitude of any potential bias	16
Interpretation	20	Give a cautious overall interpretation of results considering objectives, limitations, multiplicity of analyses, results from similar studies, and other relevant evidence	14-17
Generalisability	21	Discuss the generalisability (external validity) of the study results	16
**Other information**			
Funding	22	Give the source of funding and the role of the funders for the present study and, if applicable, for the original study on which the present article is based	2

Supplementary Table S1. Study enrollment criteria

**Inclusion Criteria**	
1	Male and female
2	Ages 12 to 70 years, inclusive
3	Existing diagnosis of ADPKD by modified Ravine criteria[a]:<br>• With family history: several cysts per kidney (3 if by sonography, 5 if by computerized tomography or MRI)<br>• Without family history: 10 cysts (by any radiologic method) per kidney and exclusion of other cystic kidney diseases
4	Ability to provide written, informed consent/assent prior to initiation of any study-related procedures, and ability, in the opinion of the investigator, to comply with all requirements of the study.
5	Total kidney volume ≥300 cc/m height by ultrasound (within 1 year prior to baseline) or ≥250 cc/m height by MRI (within 1 year prior to baseline).
**Exclusion Criteria**	
1	Any medical condition, in the opinion of the investigator, that could interfere with evaluation of the study objectives (e.g., inability to comply with MRI)
2	Current or expected (within the next six months) interventions for the treatment of ADPKD affecting kidney volume without the prior approval of the sponsor

[a] Pei Y, Obaji J, Dupuis A, et al. Unified criteria for ultrasonographic diagnosis of ADPKD. *J Am Soc Nephrol*. 2009;20:205−212.

ADPKD, autosomal polycystic kidney disease; MRI, magnetic resonance imaging.

Supplementary Table S2. Number of participants by region and country

Asia Pacific (N=422)		Europe (N=1355)		North America (N=1373)		South America (N=259)	
**Country**	**n (%)**	**Country**	**n (%)**	**Country**	**n (%)**	**Country**	**n (%)**
Australia	168 (39.8)	Belgium	32 (2.4)	Canada	211 (15.4)	Argentina	129 (49.8)
Japan	254 (60.2)	Switzerland	98 (7.2)	USA	1162 (84.6)	Brazil	130 (50.2)
		Czech Republic	42 (3.1)				
		Germany	290 (21.4)				
		Spain	51 (3.8)				
		France	88 (6.5)				
		United Kingdom	280 (20.7)				
		Italy	126 (9.3)				
		The Netherlands	68 (5.0)				
		Norway	12 (0.9)				
		Poland	88 (6.5)				
		Romania	68 (5.0)				
		Sweden	12 (0.9)				
		Turkey	100 (7.4)				

Supplementary Table S3. Types of health insurance in the study population

Insurance Type, n (%)	USA (n=1162)	Non-USA (n=2247)	Overall (N=3409)
Employer-provided health insurance	774 (66.6)	256 (11.4)	1030 (30.2)
Employer-provided health insurance and national health insurance	21 (1.8)	32 (1.4)	53 (1.6)
Employer-provided health insurance and self-insured	2 (0.2)	1 (0.0)	3 (0.1)
National health insurance	188 (16.2)	1588 (70.7)	1776 (52.1)
National health insurance and self-insured	10 (0.9)	23 (1.0)	33 (1.0)
No health insurance	58 (5.0)	137 (6.1)	195 (5.7)
Self-insured	109 (9.4)	210 (9.3)	319 (9.4)

Supplementary Figure S1. Number of participants per site

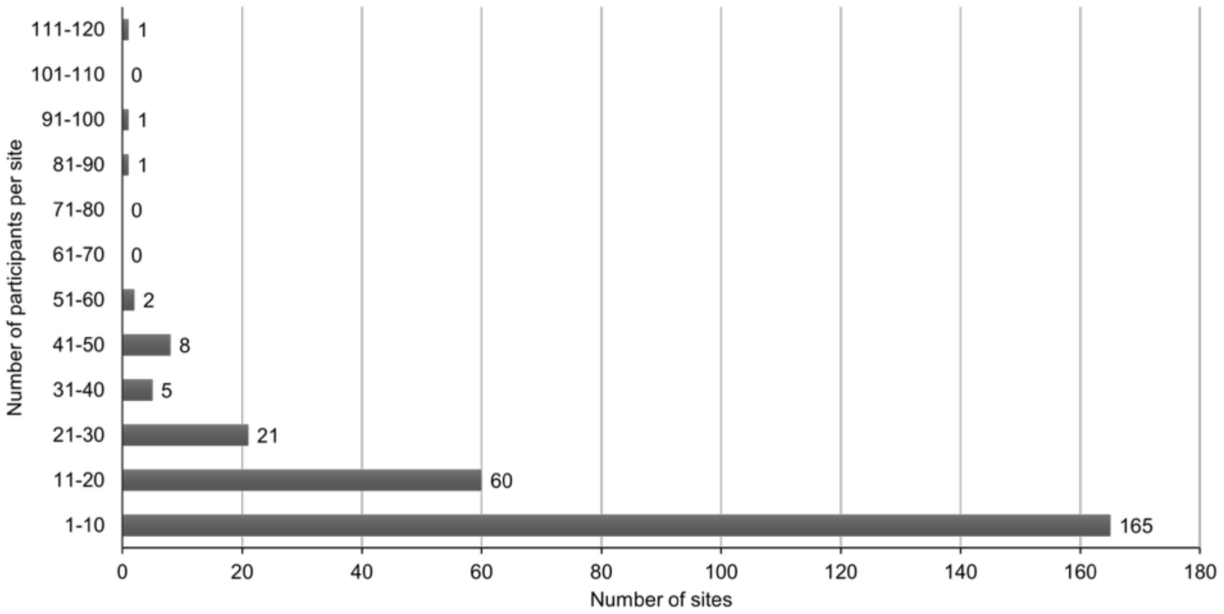

Supplementary Figure S2. Subject flow

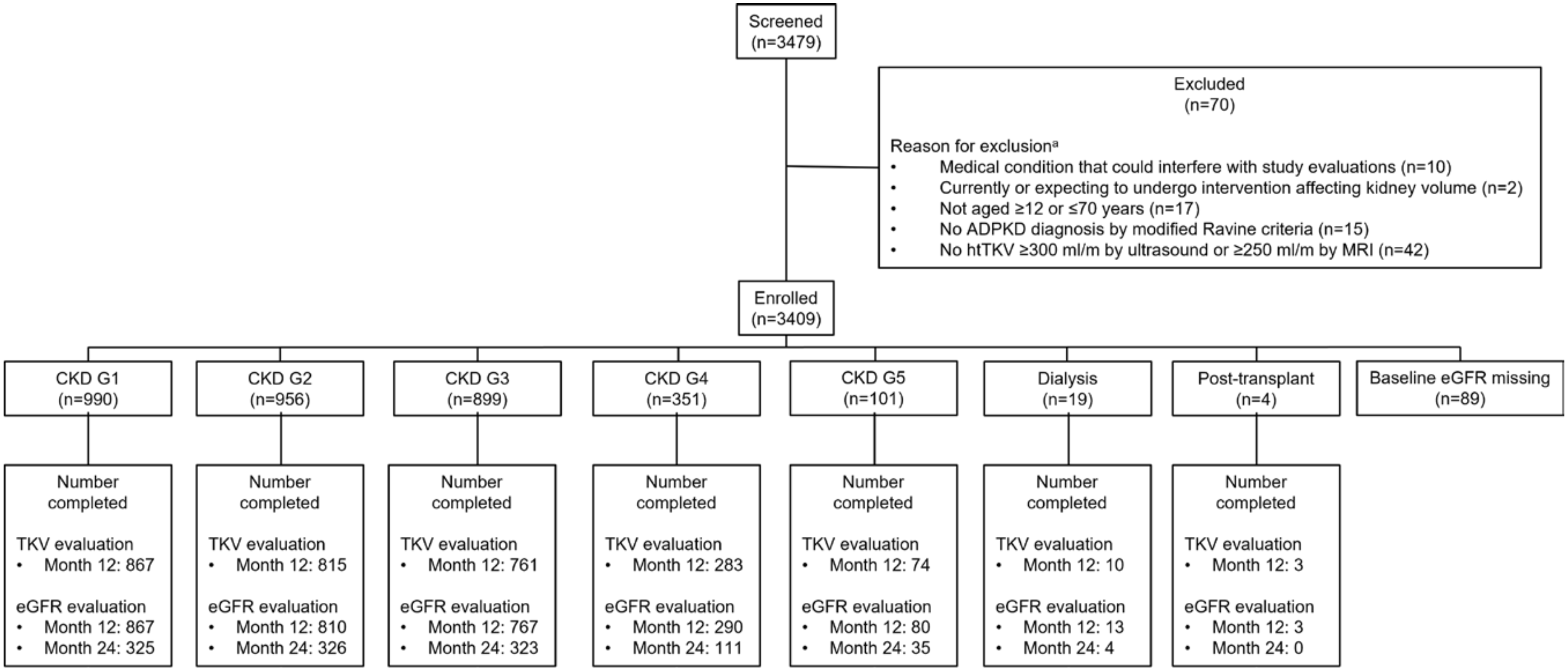

[a] Subjects could have more than one reason for exclusion.

ADPKD, autosomal dominant polycystic kidney disease; CKD, chronic kidney disease; eGFR, estimated glomerular filtration rate; htTKV, height-adjusted total kidney volume; MRI, magnetic resonance imaging; TKV, total kidney volume.

Supplementary Figure S3. Percentage of subjects reaching eGFR decline thresholds at 24 months **(a)** by baseline htTKV tertile and **(b)** by baseline Mayo imaging classification

a

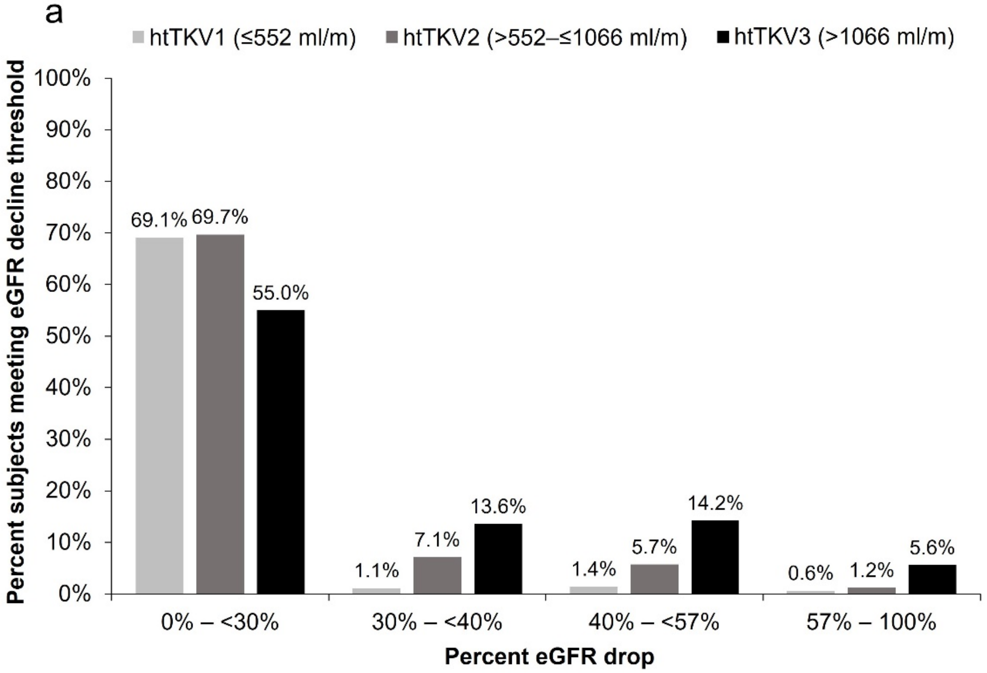

b

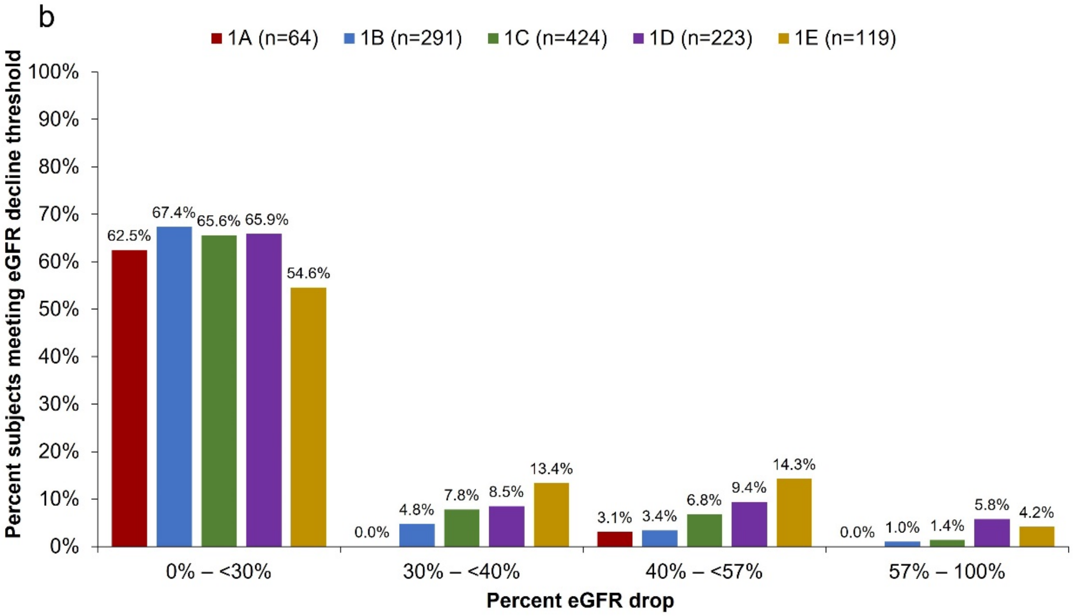

In Figure S3b, n's are the number of subjects in each baseline Mayo imaging classification who attended at the Month 24 visit.
eGFR, estimated glomerular filtration rate; htTKV, height-adjusted total kidney volume.

Supplementary Figure S4. Distribution of Mayo imaging classification by age group

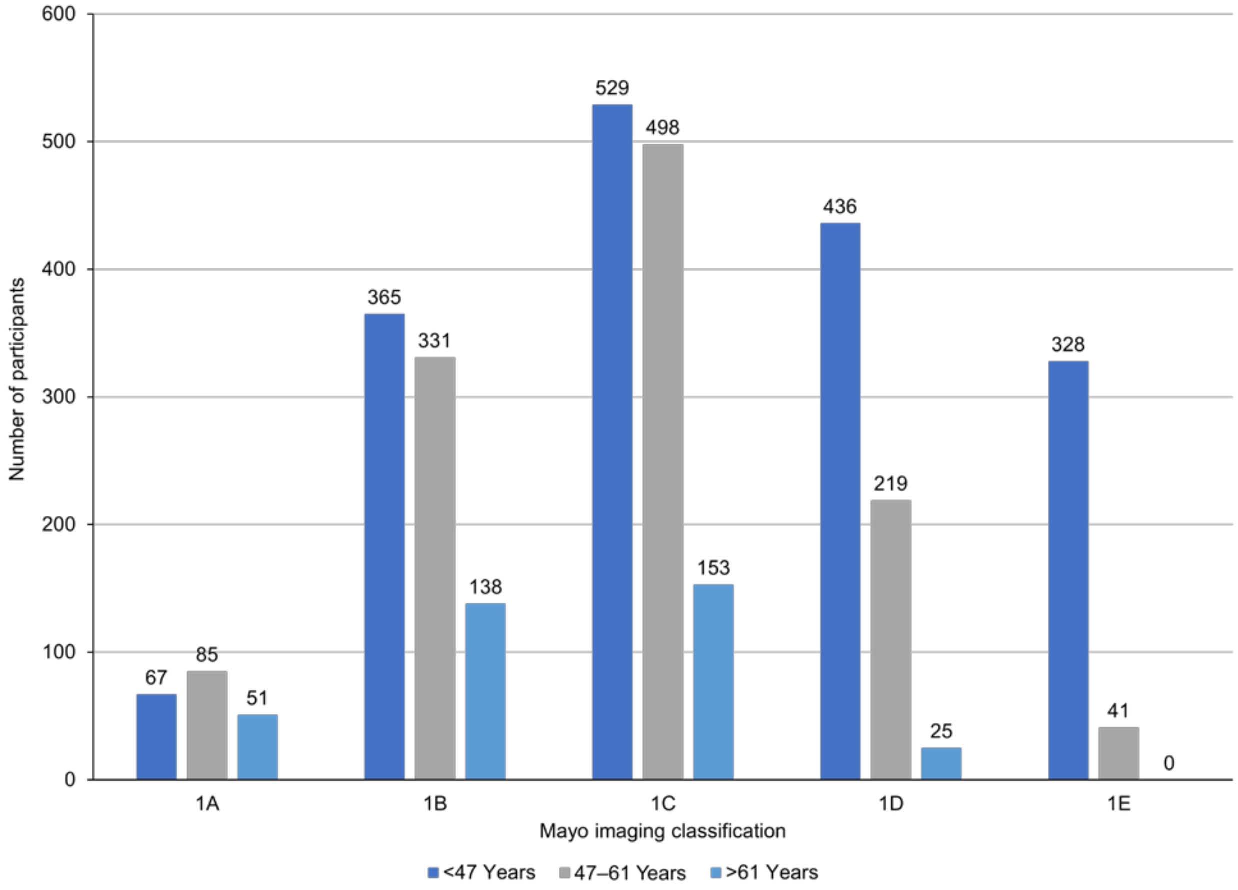

Supplementary Figure S5. One-year change in TKV. **(a)** Absolute change in TKV at Month 12 visit – individual subjects in ascending order by baseline TKV. **(b)** Subjects grouped by percent change in TKV at Month 12 visit

a

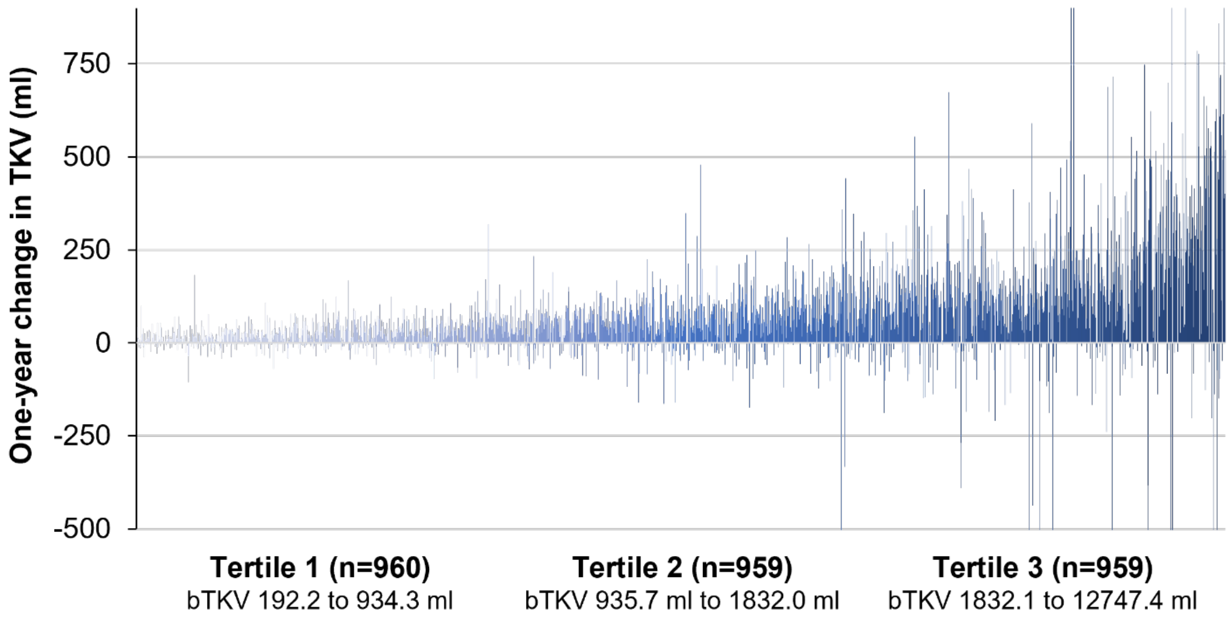

b

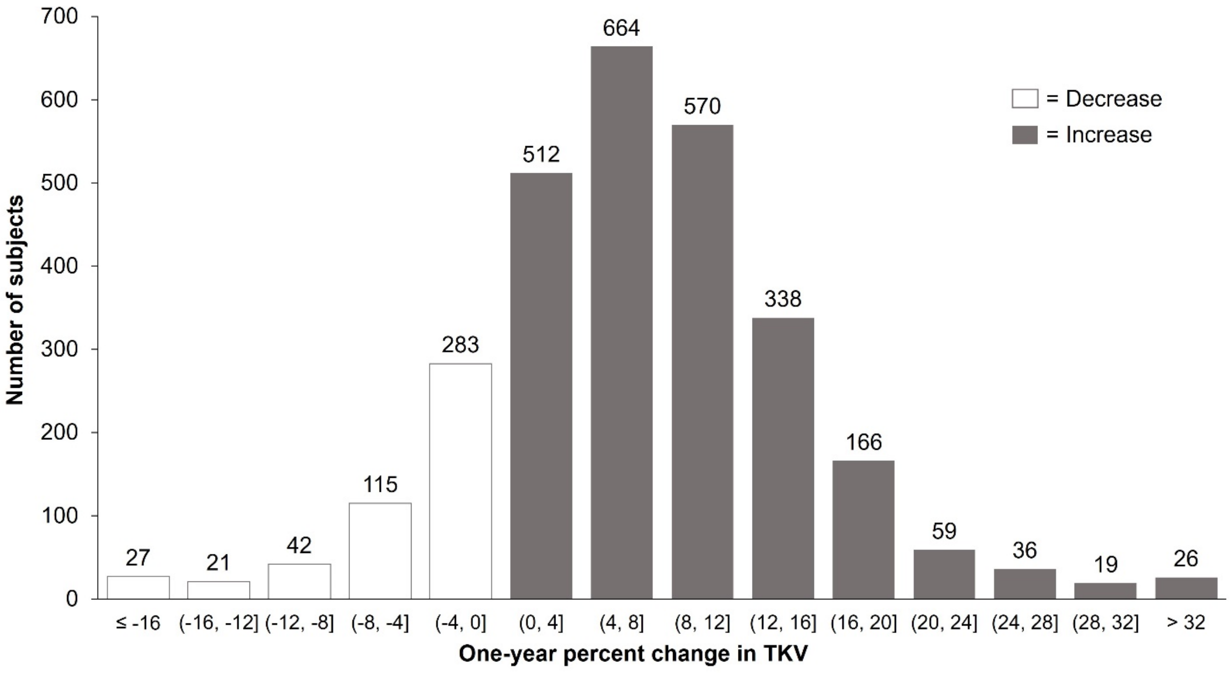

In Figure S5a, individual subjects' absolute change in TKV at the Month 12 visit are plotted in ascending order by baseline TKV from left (smallest baseline TKV) to right (largest baseline TKV). Eighteen of 2878 subjects with data are not fully displayed: 10 subjects with a decline of -1862.5, -1335.8, -1247.2, -854.0, -827.7, -696.5, -659.1, -616.8, -559.6, -510.6 ml, and 8 subjects with an increase of 776.7, 785.0, 859.6, 917.7, 972.7, 988.4, 1065.2, and 1360.1 ml during the observation period.

bTKV, baseline total kidney volume; TKV, total kidney volume.

List of Principal Investigators and Study Sites

Argentina

Ana Cusumano, Instituto de Nefrologia Pergamino, Buenos Aires; Alfredo Wasserman, MD, FEPREVA – Fundación para el Estudio, la Prevención y el Tratamiento de la Enfermedad Vascular Aterosclerótica, Buenos Aires; Rodolfo Martin, MD, Hospital Universitario Austral, Buenos Aires; Jorge de la Fuente, MD, Hospital Privado, Centro Medico de Cordoba, Córdoba; Ruben Schiavelli, MD, Hospital General de Agudos, Dr. Cosme Argerich, Buenos Aires; Hugo Beresan, MD, CEREHA S.A.D., Buenos Aires; Carlos Diaz, MD, CEMIC (Centro de Educacion Medica e Investigaciones Clinicas), Buenos Aires; Augusto Vallejos, MD, Centro de Salud Renal Junin, Buenos Aires; Pedro Alvarez, MD, Centro de Hepatología, Buenos Aires; Luis Gaite, MD, Clinica de Nefrologia, Urologia y Enfermedades cardiovasculares, Santa Fe; Nestor Garcia, MD, J. Robert Cado Foundation, Cordoba; Guillermo Rosa Diez, MD, Hospital Italiano de Buenos Aires, Buenos Aires; Maria Rial, MD, Instituto de Nefrologia Nephrology S.A., Buenos Aires.

Australia

Nigel Toussaint, Royal Melbourne Hospital, Parkville; James Mackie (formerly Bruce Pussell), Prince of Wales Hospital, Sydney; Shane Carney, John Hunter Hospital, Newcastle; Margaret Jardine, Concord Hospital, Sydney; Bruce Cooper, Royal North Shore Hospital, Sydney; Randall Faull (prev, Russ), Royal Adelaide Hospital, Adelaide; Kevin Warr, Royal Perth Hospital, Perth; Ian Fraser, Epworth Health Care, Richmond; Paul Snelling, Royal Prince Alfred Hospital, Sydney; David Packham, Reservoir Private Hospital, Melbourne; Mathew Mathew, Tasmanian Health Organisation - North Department of Medicine, Launceston; Gopala Rangan, Westmead Hospital, Westmead; Michael Suranyi, Liverpool Hospital, Liverpool.

Belgium

Olivier Devuyst, MD, Cliniques Universitaires Saint-Luc, Brussels; Patrick Peeters, MD, UZ Gent, Gent; Bert Bammens, MD, University Hospitals Leuven (UZ Leuven), Leuven.

Brazil

Irene Noronha, MD, Real e Benemerita Sociedade Portuguesa de Beneficencia, São Paulo; Elizete Keitel, MD, Irmandade Santa Casa de Misericórdia de Porto Alegre, Porto Alegre; Mário Filho, MD, Instituto de Urologia e Nefrologia de São José do Rio Preto, São José do Rio Preto; Ita Heilberg, MD, Hospital do Rim e Hipertensão - Fundação Oswaldo Ramos, São Paulo; Rosângela Milagres, MD, Hospital SOCOR, Belo Horizonte; Domingos D'Avila, MD, Hospital São Lucas da Pontificia Universidade Catolica do Rio Grande do Sul –PUC-RS, Porto Alegre; Fernando Thomé, MD, Universidade Federal do Rio Grande do Sul, Porto Alegre; Luciane Deboni, MD, Fundação Pró Rim- Fundacao de Amparo a Pesquisa em Enfermidades Renais e Metabolicas, Joinville; Miguel Riella, MD, Istituto Scribner de Ensino, Pesquisa, Ciencia e Tecnologia, Curitiba; Luiz Onuchic, MD, Hospital das Clínicas da Faculdade de Medicina da Universidade de Sao Paulo – FMUSP, São Paulo; Marcus Bastos, MD, Fundação IMEPEN, Juiz De Fora.

Canada

Paul Barre, MD, Royal Victoria Hospital, Montreal; Daniel Bichet, MD, Hospital du Sacre-Coeur de Montreal, Montreal; Stephen Chow, MD, Toronto East General Medical Centre, Toronto; Phil McFarlane, MD, PhD, FRCP (C), St. Michael's Hospital, Toronto; Alan McMahon, MD, University of Alberta, Edmonton; Hitesh Mehta, MD, Regional Kidney Wellness Center, Brampton; Sean Murphy, MD, BSc, (Hons), BSc Med, FRCP (C), Eastern Health Authority and Memorial University of Newfoundland, St. John's; York Pei, MD, FRCP (C), FACP, FASN, Toronto General Hospital University Health Network, Toronto; Daniel Sapir, MD, Ontario Nephrology Associates, Oakville; Steven Soroka, MD, Capital Health Center for Clinical Research, Halifax; Robert Ting, MD, Corporate Medical Centre, Scarborough; Murray Vasilevsky, MD, Montreal General Hospital - McGill University Health Centre, Montreal; Nadia Zalunardo,

MD (formerly C. Kit Yueng), University of British Columbia, Vancouver; George Soltys, MD, Centre de santé et de services sociaux, Greenfield Park; Monica Beaulieu, MD, St. Paul's Hospital, Vancouver.

Czech Republic

Marie Peskova, MD, Interni oddeleni, Nemocnice Ceske Budejovice a.s., Ceske Budejovice; Vladimir Tesar, MD, Vseobecna fakultni nemocnice v Praze, klinika nefrologie, Praha; Miroslav Merta, MD, Fakultni nemocnice Hradec Kralove Klinika gerontologicka a metabolicka, Hradec Kralove; Miroslav Ryba, MD, Odděěelení nefrologie a dialýzy, Krajská nemocnice Liberec a.s., Liberec; Jaroslav Tocik, MD, Interni oddeleni, Nemocnice Jihlava, Jihlava.

France

Michèle Kessler, CHU de Nancy, Vandoeuvre-lès-Nancy; Jean-Pierre Fauvel (formerly Maurice Laville), Hôpital Edouard Herriot, Lyon; Bruno Hurault de Ligny (formerly Jean-Philippe Ryckelynck), CHU de Caen, Caen; Philippe Zaoui, CHU de Grenoble, Grenoble; Claire Pouteil-Noble, Hôpital Edouard Herriot, Lyon; Hélène Leray (formerly Bernard Canaud), CHU de Montpellier, Montpellier; Pierre Trolliet, CHU Lyon Sud Service de nephrologie – transplantation-dialyse, Pierre-bénite.

Germany

Peter Gross, GWT-TUD GmbH, Dresden; Lutz Renders, MD, II. Medizinische Klinik und Poliklinik der Technischen Universität München, Munich; Kai-Uwe Eckardt, University Hospital Erlangen, Erlangen; Frank Strutz, MD, Deutsche Klinik fuer Diagnostik, Wiesbaden; Claudia Sommerer, Universitätsklinikum Heidelberg, Heidelberg; Frank Dellanna, Gemeinschaftspraxis und Dialysezentrum Karlstraße, Düsseldorf; Elke Wühl, Universitätsklinikum Heidelberg, Heidelberg; Christian Hugo, MD, University Hospital Dresden, Dresden; Klemens Budde, Campus Charité Mitte (CCM), Berlin; Hermann Haller, MD, Medizinische Hochschule Hannover, Hannover.

Italy

Gianni Cappelli (formerly Riccardo Magistroni), Azienda Ospedaliero-Universitaria Policlinico di Modena, Modena; Giovambattista Capasso, Azienda Ospedaliera Universitaria Seconda Università degli Studi di Napoli, Napoli; Ciro Esposito, MD (formerly Giuseppe Villa), Istituto Scientifico di Pavia Fondazione S. Maugeri Clinica del Lavoro e della Riabilitazione (IRCCS), Pavia; Francesco Scolari, Azienda ospedaliera Spedali Civili di Brescia - Presidio di Montichiari, Montichiari; Francesco Locatelli, Azienda Ospedaliera Ospedale di Lecco, Lecco; Antonio Santoro, Azienda Ospedaliera Universitaria Policlinico S. Orsola-Malpighi, Bologna; Salvatore Badalamenti, Istituto Clinico Humanitas, Milan; Giovanni Montini, Azienda Ospedaliera Universitaria Policlinico S. Orsola-Malpighi, Bologna.

Japan

Satoru Muto, Teikyo University Hospital, Tokyo; Shuichi Tsuruoka, Nippon Medical School Hospital, Tokyo; Eiji Ishimura, Osaka City University Hospital, Osaka; Koichi Kamura, Chiba East Hospital, Chiba; Ichiei Narita, Niigata University Medical & Dental Hospital, Niigata; Sekiya Shibazaki, Hokkaido University Hospital, Sapporo-shi; Kikuo Nutahara, Kyorin University Hospital, Tokyo; Osamu Saito, Jichi Medical University Hospital, Shimotsuke-shi; Shinichi Uchida, Tokyo Medical and Dental University, Tokyo; Kazuhiko Tsuruya, Kyushu University Hospital, Fukuoka-shi; Yoshitaka Isaka, Osaka University Hospital, Osaka; Kouju Kamata, Kitasato University Hospital, Sagamihara-shi; Naoki Kashihara, Kawasaki Medical School Hospital, Kurashiki-city; Kosaku Nitta, Tokyo Women's Medical University Hospital, Tokyo.

Netherlands

Ron Gansevoort, University Medical Centre Groningen, Groningen; Joost Drenth, MD, PhD, Radboud University Nijmegen Medical Center, Nijmegen; Johan De Fijter, MD, Leiden University Medical Center, Leiden; Robert Zietse, MD, PhD, Erasmus Medical Center Dept of Internal Medicine, Rotterdam.

Norway

Kristian Selvig, Sykehuset Buskerud Vestre Viken HF, Drammen; Haavard Aksnes, Sykehuset Innlandet Lillehammer, Lillehammer.

Poland

Kazimierz Ciechanowski, MD, Prof, Samodzielny Publiczny Szpital Kliniczny Nr 2, PUM w Szczecinie, Szczecin; Wieslaw Klatko, MD, PhD, Specjalistyczny Szpital Wojewodzki Ciechanowie, Ciechanow; Marian Klinger, MD, Prof, Akademicki Szpital Kliniczny im. Jana Mikulicza - Radeckiego, Wroclaw; Robert Malecki, MD, Miedzyleski Szpital Specjalistyczny, Warszawa; Dorota Drozdz, MD, Uniwersytecki Szpital Dzieciecy w Krakowie, Krakow; Marcin Tkaczyk, Prof, NZOZ TRI-medic, Lodz; Marzena Janas, MD, Centrum Medyczne Medyk Poradnia Nefrologiczna ze Stacja Dializ, Rzeszów; Antoni Sokalski, MD, NZOZ Centrum Medyczne AESKULAP, Radom; Ryszard Trafny, MD, Stacja Dializ NZOZ Avitum, Golub-Dobrzyn.

Romania

Mihai Voiculescu, MD, PhD, Institutul Clinic Fundeni, Bucuresti; Gabriel Bako, MD, PhD, Spitalul Clinic Municipal Dr. Gavril Curteanu Oradea, Oradea; Ovidiu Golea, MD, PhD, Spitalul Clinic Judetean de Urgenta Timisoara, Timisoara; Gabriel Mircescu, MD, PhD, Spitalul Clinic de Nefrologie Dr. Carol Davila, Bucuresti.

Spain

Miquel Hueso, Hospital Universitario de Bellvitge, Hospitalet de Llobregat; Josep Maria Campistol, Hospital Clinic de Barcelona, Barcelona; Javier Nieto, Hospital General de Ciudad Real, Ciudad Real; Carlos Santiago Guervos, Hospital General de Alicante, Alicante; Ramon Peces, Hospital Universitario la Paz, Madrid.

Sweden

Erik Fjellstedt, MD, Skane University Hospital, Malmö; Jan Wilske, Värnamo Hospital, Värnamo; Peter Barany, MD, Karolinska University Hospital, Stockholm.

Switzerland

Andreas Serra, MD, Universitäts Spital Zürich, Zürich.

Turkey

Caner Cavdar, Dokuz Eylul University Medical Faculty, Izmir; Saime Paydas, Cukurova University Medical Faculty, Adana; Soner Duman, Ege University Medical Faculty, Izmir; Neval Duman, Ankara University Medical Faculty, Ankara; Tevfik Ecder, MD, Istanbul University, Istanbul; Mustafa Cirit, Sifa University Medical Faculty, Izmir.

United Kingdom

Stewart Lambie, MBChB, FRCP, MD, Raigmore Hospital, Inverness; Iain MacPhee, BSc, PhD, BM, BCh, MRCP, FRCP, St. George's Healthcare NHS Trust, London; A. Neil Turner, PhD, FRCP, Royal Infirmary of Edinburgh, Edinburgh; Daniel Gale (formerly Patrick Maxwell), Royal Free Hospital, London; Albert Ong, BM BCh, MA, DM, FRCP, Sheffield Teaching Hospitals NHS Foundation Trust, Northern General Hospital, Sheffield; Waqar Ayub (formerly Daniel Zendher), University Hospital Coventry and Warwickshire NHS Trust, Coventry; Peter Maxwell, MD, PhD, FRCP, Belfast City Hospital Regional Nephrology Unit, Belfast; Richard Sandford, MD, PhD, MRCP, FRCP, Cambridge University Hospitals NHS Foundation Trust, Addenbrooke's Hospital, Cambridge; Detlef Bockenhauer, PhD, FRCPCH, Great Ormond Street Hospital for Children, London; John Sayer, PhD, MBChB, MRCP, Newcastle upon Tyne Hospitals NHS Foundation Trust, Newcastle Upon Tyne; Christopher Winearls, MB, ChB, PhD, FRCP, Oxford University Hospitals NHS Trust, Oxford Kidney Unit, The Churchill Hospital, Oxford; Larissa Kerecuk, MBBS, BSc, FRCPCH; Birmingham Children's Hospital, Birmingham; Nick Hateboer, MD, MB, ChB, MRCP, FRCP, Dorset County Hospital, Dorchester; Richard D'Souza, MD, FRCP, Royal Devon and

Exeter NHS Foundation Trust, Royal Devon Exeter Hospital, Exeter; David Makanjuola, MD, MBBI, MRCP (formerly Mysore Panish), Epsom and St Helier University Hospitals NHS Trust, St Helier Hospital, Carshalton; Jonathan Barratt, PhD, FRCP (formerly Nigel Brunskill), University Hospitals of Leicester NHS Trust, Leicester General Hospital, Leicester; Morwenna Wood, MA, MB, BS, MRCP, DPhil, Queen Margaret Hospital and Victoria Hospital, Kirkcaldy; Grahame Wood, MB, ChB, CCST, MD, FRCP, Salford Royal NHS Foundation Trust, Salford Royal Hospital, Salford; Sunil Bhandari, MB, ChB, MRCP, PhD, JCHMT, EDCL, FRCP, Hull and East Yorkshire Hospitals NHS Trust, Hull Royal Infirmary, Hull; Simon Davies, MD, FRCP, University Hospital of North Staffordshire NHS Trust, City General Hospital, Stoke-on-Trent; Ken Farrington, BSc, MB, Bch, MD, FRCP, East and North Hertfordshire NHS Trust, Lister Hospital, Stevenage; Kate Hillman, MB, BChir, PhD, FRCP, Central Manchester University Hospitals NHS Foundation Trust, Manchester Royal Infirmary, Manchester; David Border (formerly Colin Jones), York Teaching Hospital, NHS Foundation Trust, York; Johann Nicholas, MB, ChB, The Royal Wolverhampton Hospitals NHS Trust, New Cross Hospital, Wolverhampton; Matthew Howse, MBBS, MRCP, MD (formerly Pearl Pai), The Royal Liverpool and Broadgreen University Hospitals NHS Trust, Royal Liverpool University Hospital, Liverpool; Robert Lewis, MD, Portsmouth Hospitals NHS Trust, Queen Alexandra Hospital, Portsmouth; David Reaich, MB, ChB, MD, FRCP, South Tees Hospitals NHS Foundation Trust, The James Cook University, Middlesbrough; Moin Saleem, MBBS, MRCP, PhD, FRCP, University Hospitals Bristol NHS Foundation Trust, Bristol Royal Hospitals for Children, Bristol; Laurie Solomon, BA, BChir, MA, MB, MRCP, MD, FRCP, Lancashire Teaching Hospitals NHS Foundation Trust, Royal Preston Hospital, Preston; John Stoves, MB ChB, BSc, MD, FRCP, Bradford Teaching Hospitals NHS Foundation Trust, St. Luke Hospital, Bradford; Sumith Abeygunasekara, MBBS, MD, FRCP, Mid Essex Hospital Services NHS Trust, Broomfield Hospital, Chelmsford.

United States

Muralidhar Acharya, MD, Outcomes Research International Inc., Spring Hill, FL; Mario Almeida, MD, ARCAMED LLC, Miami, FL; Balaji Athreya, MD, Pioneer Valley Nephrology, Inc., Holyoke, MA; Jon Blumenfeld, MD, The Rogosin Institute, New York, NY; Chandra Chandran, MD, St. Joseph's Regional Medical Center, Paterson, NJ; Arlene Chapman, MD, Emory University Hospital, Atlanta, GA; Chaim Charytan, MD, Nephrology Associates, PC, Flushing, NY; Teresa Levitski- Heikkila, MD (formerly Daniel Louvar), Sanford Clinic, Fargo, ND; Andrew Cortez, DO, AKDHC, Inc., Peoria, AZ; Richard Cottiero, MD, Hypertension Nephrology, Providence, RI; Neera Dahl, MD, PhD, Yale University School of Medicine, New Haven, CT; Francis Dumler, MD, William Beaumont Hospital, Royal Oak, MI; Mohamed El-Shahawy, MD, MPH, MHA, Academic Medical Research Institute, Los Angeles, CA; Stephen Fadem, MD, Kidney Associates, PLLC, Houston, TX; Margaret Franger, MD, Center for Kidney Care, Hainesport, NJ; Michael Germain, MD, Western New England Renal Transplant Associates, PC, Springfield, MA; David Gillum, MD, Western Nephrology & Metabolic Bone Disease, PC, Arvada, CO; Seth Goldberg, MD, Washington University in St. Louis, St. Louis, MO; Simin Goral, MD, University of Pennsylvania Health System, Philadelphia, PA; John Middleton, MD (formerly Arthur Greenburg); Duke University, Durham, NC; Adetola Haastrup, MD, Altru Health System, Devils Lake, ND; Rekha Halligan, MD, PhD, Bayview Nephrology, Inc, Erie, PA; Azzour Hazzan, MD, North Shore University Hospital, Great Neck, NY; Aamir Jamal, MD, North America Research Institute, Azusa, CA; Kotagal Kant, MD, University of Cincinnati, Cincinnati, OH; Kianoosh Kaveh, DO, Coastal Nephrology Associates Research Center, LLC, Port Charlotte, FL; Abid Khan, MD, Abid Khan, MD, Ontario, CA; Nelson Kopyt, DO, Northeast Clinical Research Center, LLC, Bethlehem, PA; Lawrence Lehrner, MD, Kidney Specialists of Southern Nevada, Las Vegas, NV; Lionel Mailloux, MD, NS/LIJ Long Island Hypertension & Nephrology, Port Washington, NY; Roberto Mangoo-Karim, MD, Gamma Medical Research, Mission, TX; Bhasker Rai Mehta, MD, Arlington Nephrology, Arlington, TX; Roy Allan Jhagroo, MD (formerly Sanjeev Shah), University of Wisconsin Kidney Clinic, Madison, WI; Patrick Nachman, MD, University of North Carolina at Chapel Hill, Chapel Hill, NC; Jesus Navarro, MD, Genesis Clinical Research, Tampa, FL; A. Kaldun Nossuli, MD, A. Kaldun Nossuli, MD PA, Bethesda, MD; Jennifer Othersen, MD, WJB Dorn VA Medical Center, Columbia, SC; Amy Pai, PharmD, Albany College of Pharmacy and Health Sciences, Albany, NY; Ronald Perrone, MD, Tufts Medical Center/Division of Nephrology, Boston, MA; Joseph Pitone, DO, Nephrology and

Hypertension Associates of NJ, Voorhees, NJ; Khalil Rahman, MD, Research Associates of Central Kentucky, Lexington, KY; Daniel Ries, MD, Twin Cities Clinical Research, Brooklyn Center, MN; Raul Rodelas, MD, Arizona Kidney Disease & Hypertension Medical Research Services, LLC, Peoria, AZ; Dennis Ross, MD, Kansas Nephrology Research Institute, LLC, Wichita, KS; Michael Ruddy, MD, Princeton Hypertension-Nephrology Associates, LLC., Princeton Junction, NJ; Rebecca Schmidt, DO, West Virginia University, Morgantown, WV; Robert Schrier, MD, University of Colorado Denver, Aurora, CO; David Scott, MD, Clinical Research Development Services, Rosedale, NY; Muhammad Shakeel, MD, Nephrology and Internal Medicine of Anderson, Anderson, SC; Arnold Silva, MD (formerly Amit Sharma), Pacific Renal Research Institute, Meridian, ID; Shayan Shirazian, MD, Winthrop University Hospital, Mineola, NY; Robert Cohen, DO (formerly Bhupinder Singh), Southwest Clinical Research Institute, LLC, Tempe, AZ; Mark Smith, MD, Kidney Care Associates, LLC, Augusta, GA; Wolfgang Weise, MD, University of Vermont, Burlington, VT; Renuka Sothinathan, MD, CPI, Clinical Research and Consulting Center, LLC, Fairfax, VA; Leslie Steed, MD, Northwest Renal Clinic, Inc, Portland, OR; Theodore Steinman, MD, Beth Israel Deaconess Medical Center, Boston, MA; Jeremy Taylor, MD, University of Rochester Medical Center, Rochester, NY; Vicente Torres, MD, PhD, Mayo Clinic, Rochester, MN; Freemu Varghese, MD, FACP, Diagnostic Clinic of Houston, Houston, TX; Manuel Velasquez, MD, The George Washington University Medical Faculty Associates, Washington, DC; Rocco Venuto, MD, Erie County Medical Center, Buffalo, NY; Steven Vicks, MD, Sierra Nevada Nephrology Consultants, Reno, NV; Ahmad Tuffaha, MD (prev. Wang), University of Kansas Medical Center, Kansas City, KS; Tariq Shafi, MD, MHS (formerly Terry Watnick), Johns Hopkins Bayview Medical Center, Baltimore, MD; Marc Weiner, MD, Hypertension & Kidney Specialists, Lancaster, PA; Terry Watnick, MD (formerly Matthew Weir), University of Maryland, Baltimore, MD; Louis Raymond, MD, Regional Medical Clinic – Nephrology, Rapid City, SD; Pablo Pergola, MD, PhD, Clinical Advancement Center, PLLC, San Antonio, TX; Jorge Serje, MD, NY Medical Care PC, Brooklyn, NY; Jeffrey Ryu, MD, NKDHC – Kantor Nephrology Research, Las Vegas, NV; Keith Bellovich, DO, Renaissance Renal Research Institute, LLC, Detroit, MI; Cluny Lefevre, DO, Night and Day, New York, NY; Jeffrey Giullian, MD, South Denver Nephrology Associates, Denver, CO; Piotr Lazowski, MD, South Shore Nephrology, P.C., Plymouth, MA; Mohamed Sekkarie, MD, Nephrology and Hypertension Associates, Bluefield, WV; Irina Barash, MD, New York University School of Medicine, New York, NY; Sorana Hila, MD, Drs. Hecht, Bass, Rosen and Schwartz PA., Wheaton, MD; Nicole Stankus, MD, University of Chicago, Chicago, IL; Melchiore Vernace, MD, Nephrology-Hypertension Specialists, Doylestown, PA; Mario Belledonne, MD, Biolab Research LLC, Rockville, MD; Shirisha Bodana, DO (formerly Fred Husserl), Ochsner Clinic Foundation, New Orleans, LA; Anjay Rastogi, MD, UCLA, Los Angeles, CA; Fahd Al-Saghir, MD, Michigan Kidney Consultants PC, Pontiac, MI; Avinash Vallurupalli, DO, Tarrant Nephrology Associates, Fort Worth, TX; Lavinia Negrea, MD (formerly Bruce Berger), University Hospitals Case Medical Center, Cleveland, OH; John Buerkert, MD (formerly Tarek Sobeih), Columbia Nephrology Associates, P.A., Columbia, SC; Anil Agarwal, MD, The Ohio State University Division of Nephrology, Columbus, OH; Pawan Gupta, MD, Altoona Kidney, Altoona, PA; James Dilley, MD, Brookview Hills Research Associates, LLC, Winston-Salem, NC; G. Edward Newman, MD, Knoxville Kidney Center, PLLC, Knoxville, TN; Than Oo, MD, Nephrology Center, PC, Kalamazoo, MI; Stephan Bart, MD, Accelovance, Rockville, MD; Daniel Brune, MD, Accelovance, Peoria, IL; Laura Helman, DO, Accelovance, Mishawaka, IN; Darren Farnesi, MD (formerly Martin Kabongo), Accelovance, San Diego, CA; Murray Kimmel, DO, Accelovance, Melbourne, FL; Michael Koren, MD, Jacksonville Center for Clinical Research, Jacksonville, FL; Jayant Kumar, MD, Renal Medicine Associates, Albuquerque, NM; Shaukat Ali, MD, Four Rivers Clinical Research, Inc., Paducah, KY.
